# Modelling of intensive care unit (ICU) length of stay as a quality measure: a problematic exercise

**DOI:** 10.1186/s12874-023-02028-x

**Published:** 2023-09-14

**Authors:** John L. Moran, Graeme J. Duke, John D. Santamaria, Ariel Linden, the Australian & New Zealand Intensive Care Society (ANZICS) Centre for Outcomes & Resource Evaluation (CORE), David Pilcher, Paul Secombe, Ed Litton, Craig Carr, Johnny Millar, Tamishta Henson, Sue Huckson, Shaila Chavan, Jennifer Hogan

**Affiliations:** 1 Department of Intensive Care Medicine, The Queen Elizabeth Hospital, Woodville, Australia; 2 Department of Intensive Care, Eastern Health, Box Hill, Australia; 3 Department of Critical Care Medicine, St Vincent’s Hospital (Melbourne), Fitzroy, Australia; 4 Linden Consulting Group, LLC, San Francisco, CA USA; 5 Australian & New Zealand Intensive Care Society (ANZICS) Centre for Outcomes & Resource Evaluation (CORE), Melbourne, Australia

**Keywords:** Length of stay, Intensive care, Generalised linear mixed model, Linear mixed model, Risk adjusted, Quality metric, Rank confidence sets

## Abstract

**Background:**

Intensive care unit (ICU) length of stay (LOS) and the risk adjusted equivalent (RALOS) have been used as quality metrics. The latter measures entail either ratio or difference formulations or ICU random effects (RE), which have not been previously compared.

**Methods:**

From calendar year 2016 data of an adult ICU registry-database (Australia & New Zealand Intensive Care Society (ANZICS) CORE), LOS predictive models were established using linear (LMM) and generalised linear (GLMM) mixed models. Model fixed effects quality-metric formulations were estimated as RALOSR for LMM (geometric mean derived from log(ICU LOS)) and GLMM (day) and observed minus expected ICU LOS (OMELOS from GLMM). Metric confidence intervals (95%CI) were estimated by bootstrapping; random effects (RE) were predicted for LMM and GLMM. Forest-plot displays of ranked quality-metric point-estimates (95%CI) were generated for ICU hospital classifications (metropolitan, private, rural/regional, and tertiary). Robust rank confidence sets (point estimate and 95%CI), both marginal (pertaining to a singular ICU) and simultaneous (pertaining to all ICU differences), were established.

**Results:**

The ICU cohort was of 94,361 patients from 125 ICUs (metropolitan 16.9%, private 32.8%, rural/regional 6.4%, tertiary 43.8%). Age (mean, SD) was 61.7 (17.5) years; 58.3% were male; APACHE III severity-of-illness score 54.6 (25.7); ICU annual patient volume 1192 (702) and ICU LOS 3.2 (4.9). There was no concordance of ICU ranked model predictions, GLMM versus LMM, nor for the quality metrics used, RALOSR, OMELOS and site-specific RE for each of the ICU hospital classifications. Furthermore, there was no concordance between ICU ranking confidence sets, marginal and simultaneous for models or quality metrics.

**Conclusions:**

Inference regarding adjusted ICU LOS was dependent upon the statistical estimator and the quality index used to quantify any LOS differences across ICUs. That is, there was no “one best model”; thus, ICU “performance” is determined by model choice and any rankings thereupon should be circumspect.

**Supplementary Information:**

The online version contains supplementary material available at 10.1186/s12874-023-02028-x.

## Introduction

The use of intensive care unit (ICU) length of stay (LOS) and its (covariate) risk adjusted equivalent (RALOS), similar to risk adjusted mortality, as a quality metric and a proxy for costs has a long history [1–3]. Systematic reviews of variables predicting LOS [4] and statistical estimators of RALOS have appeared [5, 6], albeit caveats about such an endeavour, particularly with respect to individual patients, have been expressed [7, 8]. The relationship between observed LOS and the expected RALOS of a cohort of ICUs may be formulated as a difference, observed minus expected LOS (OMELOS [9]) or as a ratio (the risk adjusted LOS ratio, RALOSR [10]), with corresponding confidence intervals (CI) and displayed in a ranked “caterpillar” plot [11]. ICU LOS ranking uncertainty may be also addressed with respect to a single ICU (versus all other ICUs) or simultaneously, across all ICUs [12]; these being two different estimands [13].

The purpose of this paper is to address these themes by way of a particular estimator of RALOS, the generalized linear mixed model (GLMM [6, 14]) compared with the more familiar linear mixed model (LMM, [6, 10]). Becker et al. have cautioned regarding the misalignment between statements of hypotheses in terms of non-transformed variables (for instance, raw ICU LOS) and the transformed data (log ICU LOS) used to test them [15]. That is, inference on the transformed (log) scale does not equate with inference on the original scale [16–18]; back-transformation via exponentiation from the log yields a geometric (mean) value. This difference was resolved by appropriate choice of family and link functions within the GLMM framework. The monotonicity or otherwise between the RALOSR in the (mean) ranked arithmetic (GLMM) or geometric (LMM) metric across ICUs was determined and the impact of formal ranking procedures [12] was examined.

## Methods

### Ethics statement

Access to the data was granted by the Australian and New Zealand Intensive Care Society (ANZICS) Centre for Outcomes & Resource Evaluation (CORE) Management Committee in accordance with standing protocols; local hospital (The Queen Elizabeth Hospital) Ethics of Research Committee waived the need for patient consent to use their data in this study. The dataset was anonymized before release to the authors by ANZICS CORE, custodians of the database. The dataset is the property of the ANZICS CORE and contributing ICUs and is not in the public domain. Access to the data by researchers, submitting ICUs, jurisdictional funding bodies and other interested parties is obtained under specific conditions and upon written request [19].

### Data management

Data was accessed from the ANZICS Adult Patient Database [20], in this instance for calendar year 2016, and processed as previously detailed [21]. Individual ICUs were anonymized, but for purposes of data management and illustration, were given non-identifying integer values.

### Statistical analysis

The modelling approach was to use a parsimonious set of predictor variables and their interactions similar to a previous paper utilizing data from the ANZICS Adult Patient Database [21]; no automated routine for covariate selection, such as stepwise regression, was used. The primary focus was the prediction of RALOS and not on coefficient interpretation, albeit subscribing to a data- not algorithmic-modeling scenario, as defined in Breiman 2001 [22].

1. Prediction of ICU LOS
  - GLMM: this was undertaken using the Stata™ (Version 17) module “meglm” (gaussian family, log link) with ICU site as a random intercept and ICU LOS (in days, the original scale of the dependent variable, calculated from date-stamped hour & minute electronic records) as the dependent variable.
  - Predicted LOS was established as “fitted” values including site specific random effect (RE) and for the fixed part of the model (FE).
  - Performance sensitivity analysis was undertaken using split sample estimation (60%) / validation (40%) technique, based upon random allocation of site as a stratum.
  - R-squared (*R*^2^), at the patient and ICU level, was calculated as the square of the (product-moment) correlation coefficient of LOS versus model predictions. With respect to *R*^2^: at the patient level values of 20–28% and at the ICU level, 50–70% have been previously found for predictive models [5, 7].
  - Different GLMM family and link combinations were also used, based on the distribution of the LOS (positive integer values with skewed distribution): gamma family and Poisson family with log link.
  - OMELOS was calculated as observed LOS minus RALOS, the latter from the “meglm” output. For each ICU, point estimates and CI were calculated using the “mean” and “bca” (bias corrected and accelerated bootstrap [23]) commands provided by Stata™.
  - Ratios of observed LOS and RALOS (risk adjusted LOS ratio, RALOSR [10]) were also computed using the “ratio” and “bca” bootstrap (1000 repetitions) commands of Stata™.
  - Visualisation of ICU LOS and model predictions was performed using kernel density plots [24] (smoothed histograms) in Stata™.
2. ICU LOS as a quality metric
  - This was undertaken using fixed effects model predictions following Straney et al. [10], not including site specific RE, to avoid adjusting for what was desired to establish, that is, ICU performance.
  - As well as the outputs from the GLMM above, a LMM was estimated, as “mixed” within Stata™ Version 17, with the same variables as with the GLMM model, ICU site as a random intercept and the dependent variable transformed to the log scale (log(LOS)), again following Straney [10].
    i. Normality of log ICU LOS was tested computationally and graphically using the user-written Stata module “qctest” [25].
    ii. Predictions (log(LOS)) were estimated from both the fitted (RE) and fixed (FE) parts of the model.
    iii. for “mixed”, ICU RALOSRs were established using a user-written (jlm) “ratio” command to compute the ratio of the geometric means of the LOS and the RALOS, which was subsequently bootstrapped to estimate “bca” CI.
  - As a sensitivity analysis, RE and their standard errors (SE) were predicted at the ICU level from both the “meglm” and “mixed” models and 95%CI were calculated for the point estimates (± 1.96*SE) [26].
3. Model specification was checked using:
  - Covariate selection was undertaken using information criteria; Akaike (AIC) and Schwartz’s Bayesian (BIC) criteria [27]. Further details are provided in the Supplementary file (“Stata command syntax and model specification”, P 2/3).
  - Residual analysis: for the GLMM, deviance and Anscombe; for the LMM, conventional and standardised residuals [26].
  - *R*^2^ estimates at the patient and ICU level (see above)
4. OMELOS, RALOSR and ICU RE displays were produced using the Stata user-written module “forest” (through “metan” V 4.05, 29^th^ November 2021: [28]); metric point estimates were ranked in the displays.
5. Using the point estimate and SE from OMELOS, RALOSR and ICU RE estimates, displays of rank confidence sets, both marginal and simultaneous, were produced using the R statistical package “csranks”[29].

## Results

### Details of cohort

The initial data base for the calendar year 2016 consisted of 94,361 adult patients from 125 ICUs with median annual patient number of 524 (25th percentile 328, 75^th^ percentile 1028, minimum 152 and maximum 2887). Patient demographics are displayed in Table 1.

**Table 1:** Cohort demographics

Variable	Value
Age: years	61.7 (17.5)^a^
Pre ICU hospital days	1.5 (2.9)^a^
	0.4 (0.2–1.1)^b^
ICU annual volume	1192 (702)^a^
	1063 (570–1859)^b^
APACHE III score	54.6 (25.7)^a^
ICU LOS: days^d^	3.2 (4.9)^a^
	1.8 (0.9–3.6)^b^
	1.9 (2.6)^c^
Hospital LOS: days	11.8 (13.2)^a^
	8.1 (4.6–14.0)^b^
ANZICS ROD	0.017 (0.005–0.073)^b^
Gender: male	58.3% (54,975)
Died ICU	6.5% (6105)
Died hospital	8.8% (8280)
Ventilated: day 1	43.5% (41,003)
Acute renal failure	5.0% (4693)
Cardiac arrest: pre-ICU	3.4% (3076)
Treatment limitation	5.2% (4772)
Hospital Classification	
Metropolitan	16.9% (15,939)
	520 (396–1030)^b^
Private	32.8% (30,983)
	983 (579–1296)^b^
Rural / Regional	6.4% (6075)
	326 (226–449)^b^
Tertiary	43.8% (41,364)
	1500 (1063–2105)^b^

*ICU* Intensive Care Unit, *LOS* Length of stay, *ROD* Patient risk of death estimate

^a^Mean(SD). ^b^Median(Inter quartile range). ^c^Geometric mean(SD). ^d^Truncated at 180 days

The patient variables used to model ICU LOS were age (and its square), APACHE III score (and its square), ANZICS risk of death score (log), pre-ICU days; death in ICU [30], acute renal failure, treatment limitation, cardiac arrest pre-ICU and mechanical ventilation on day 1 of ICU (as binary variables, 1/0); hospital ICU classification (4 level categorical); collapsed APACHE III categorical variables for surgical and medical diagnoses (30 level; see Supplementary files: Appendix 1, Table 1). Multiple variable interactions were utilised in modelling; Stata command syntax for both GLMM and LMM is given in Supplementary files: Appendix 1, page 2. GLMM and LMM models converged satisfactorily with a total patient number of 87,980, representing, for complete case analysis, a missing data fraction of 9%; no multiple imputation was undertaken.

### Modelling approaches

#### Generalised linear mixed model (GLMM)

The GLMM converged after 124 iterations, requiring the built-in Stata™ maximization option “difficult” and the non-default BFGS (Broyden–Fletcher–Goldfarb–Shanno) algorithm. Model coefficients are displayed in Supplementary files: Appendix 1, Table 2. Residual (deviance and Anscombe) analysis was acceptable and predicted ICULOS values are shown in Table 2.

**Table 2:** Predicted values of ICU length of stay; *n* = 87,980

Variable	Minimum	p25	p50	mean	p75	Maximum
ICU days	0.003	0.944	1.837	3.214	3.598	126.913
GLMM: fitted (RE), days	0.141	1.617	2.561	3.177	4.072	82.029
GLMM: fixed, days	0.08	0.979	1.992	3.612	4.275	66.768
OMELOS: fixed, days	-62.534	-1.925	-0.004	-0.398	1.172	123.032

p25, p50 & p75; 25^th^, 50^th^ and 75^th^ percentiles

*GLMM* Generalised linear mixed model, *RE* Random effects included in estimates, *Fixed* No random effects included in estimates, *OMELOS* Observed minus expected length of stay

**Fig. 1:**
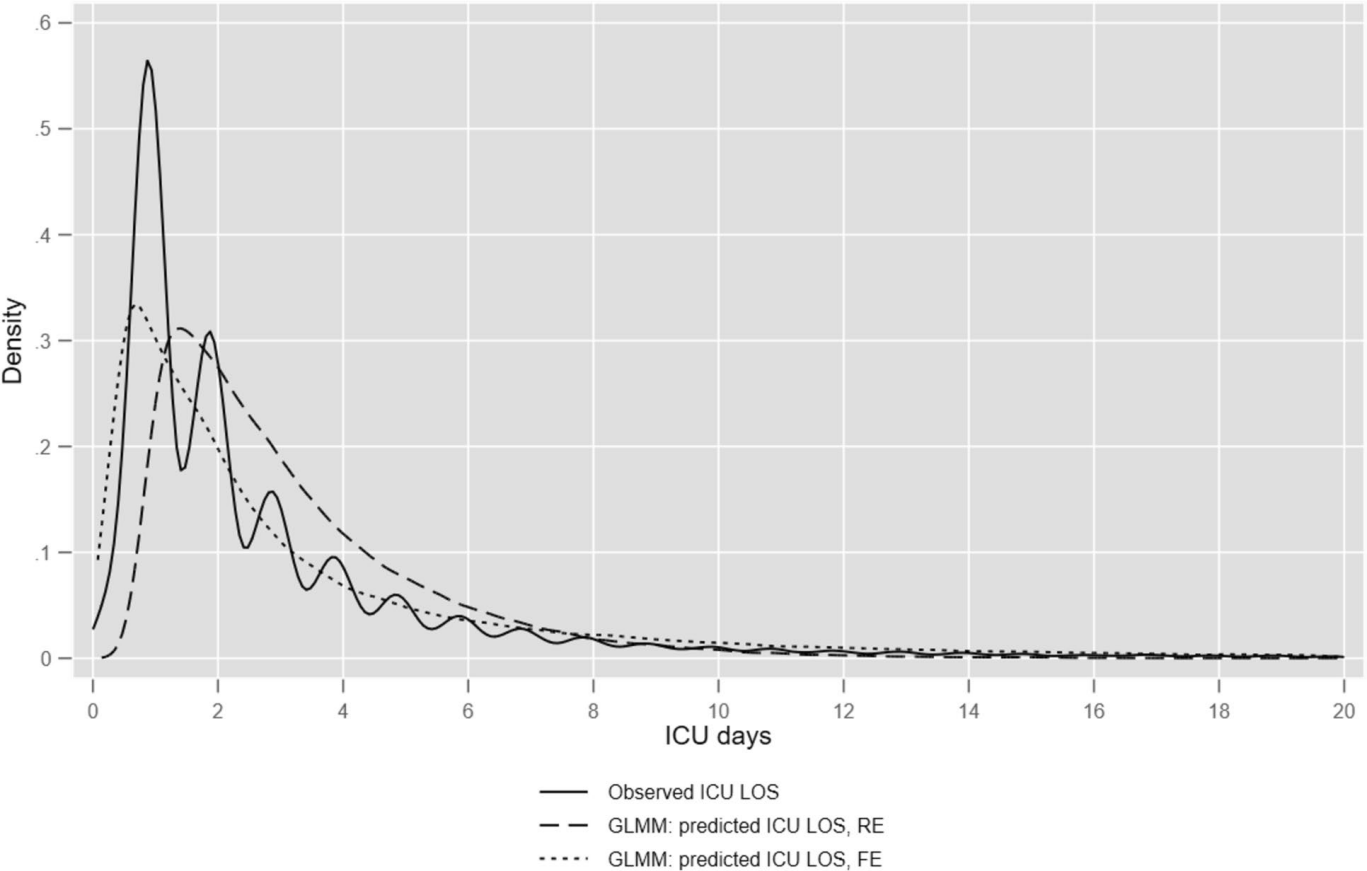
Kernel density plots of observed ICU LOS & GLMM predictions (RE and FE), truncated at 20 days

The GLMM predictions compared with ICU LOS are displayed in Fig. 1 using kernel density plots.

For the RE model, the split-sample sensitivity analysis yielded patient *R*^2^ (predicted versus observed ICULOS) of 0.19 (development set, *n* = 48,015, 75 ICUs) and 0.21 (validation set, *n* = 39,965, 49 ICUs). For the whole estimation sample, *n* = 87,980, patient and ICU *R*^2^ were 0.20 and 0.85 respectively.

Two different GLMM family and link combinations, gamma family and Poisson family with log link, failed to converge.

#### Linear mixed model (LMM)

The LMM converged rapidly; model coefficients are displayed in Supplementary files: Appendix 1, Table 3. Residual (conventional and standardised) analysis was acceptable and predicted ICU LOS values in the log-metric are shown in Table 3. Log-ICU LOS was not normally distributed as per the “qctest” Stata module.

**Table 3:** Predicted ICU length of stay (log metric) values; *n* = 87,980

Variable	Minimum	p25	p50	mean	p75	Maximum
Log ICU days	-5.663	-0.057	0.608	0.659	1.28	4.844
LMM: fitted (log)	-1.617	0.26	0.632	0.659	1.003	2.798
LMM: fixed (log)	-1.663	0.258	0.633	0.651	0.989	2.933

p25, p50 & p75; 25^th^, 50^th^ and 75^th^ percentiles

*LMM* Liner mixed model, *Fitted* Random effects included in estimates, *Fixed* No random effects Included in estimates

For the RE model (log metric), the split-sample sensitivity analysis yielded patient *R*^2^ (predicted versus observed ICULOS) of 0.30 (*n* = 48,015) and 0.28 (*n* = 39,965). For the whole estimation sample, *n* = 87,980, patient and ICU *R*^2^ were 0.29 and 0.96 respectively.

**Fig. 2:**
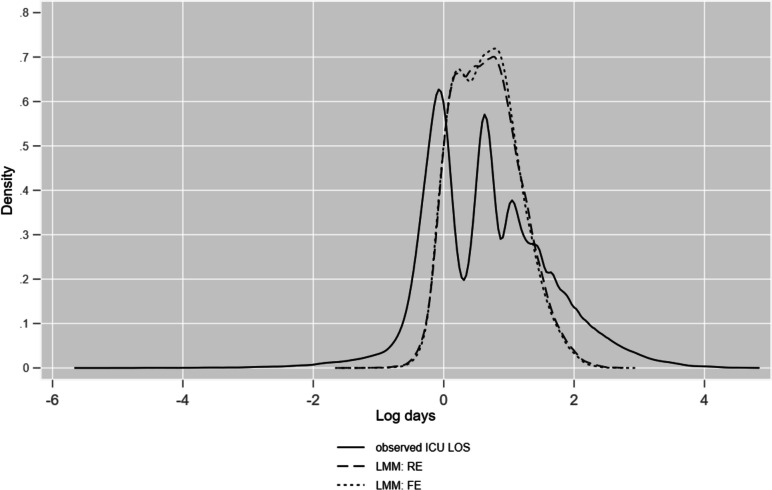
Kernel density plots of observed ICU LOS and LMM predictions; log metric

The LMM log predictions compared with log (observed) ICU LOS are seen in Fig. 2 using kernel density plots.

**Fig. 3:**
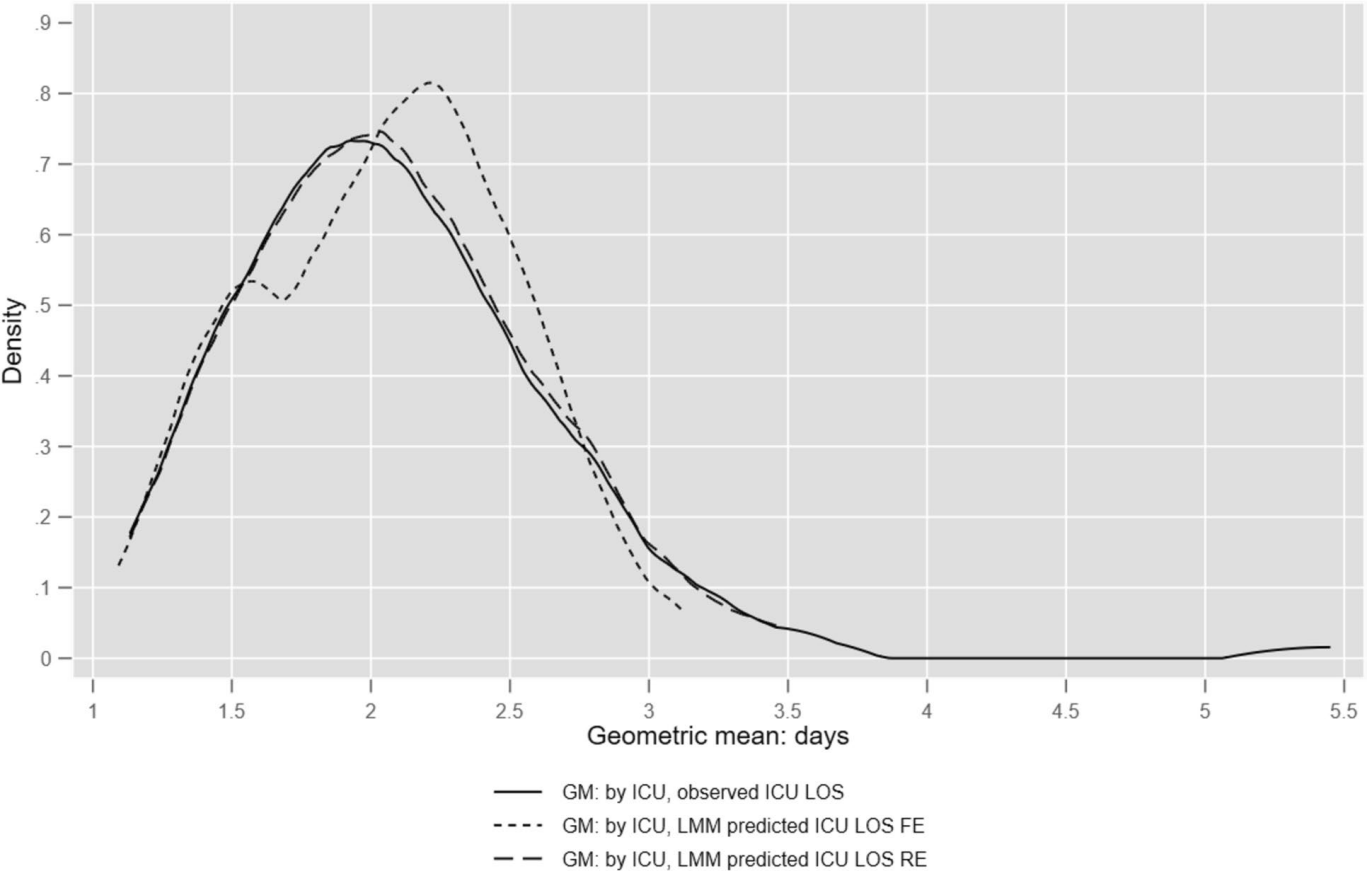
Kernel density plots of geometric means (GM) by ICU for observed ICU LOS and LMM predictions

Similarly, ICU LOS geometric means are plotted in Fig. 3 for raw ICU LOS and LMM predictions (fixed and random effects).

For the whole estimation sample, *n* = 87,980, ICU *R*^2^ was 0.38 and 0.88 for the fixed and random effects LMM models.

### Quality metrics: tertiary ICUs used as exemplars

#### RALOSR FE: GLMM vs LMM

**Fig. 4:**
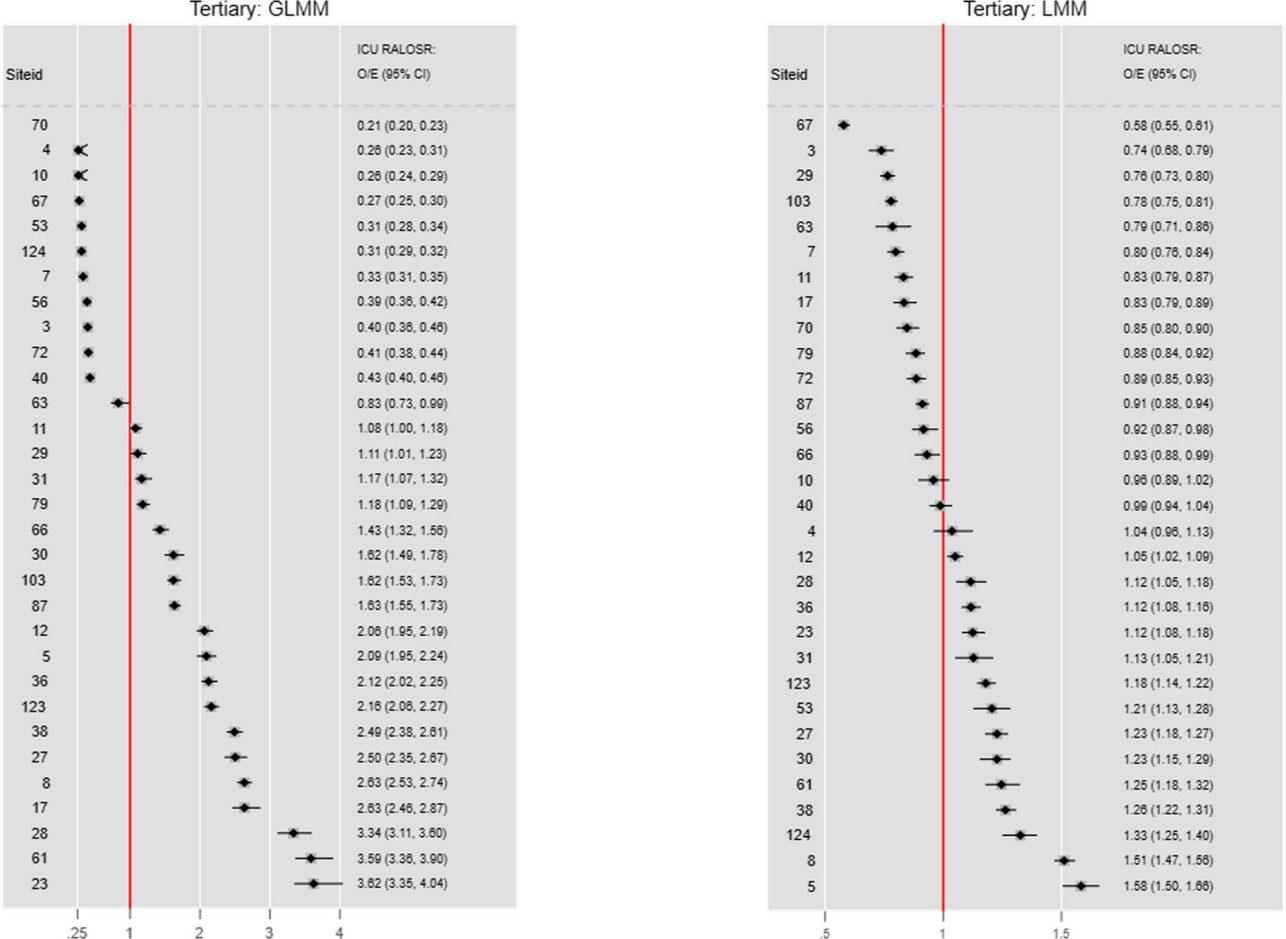
RALOSR for fixed effects, GLMM versus LMM

The combined graph (Fig. 4) shows the ratio changes across the spread of ICUs, but there was no concordance of ICU rankings between the two estimators, albeit the comparison is between the arithmetic and geometric LOS predictions. For the GLMM, lower RALOSR 95% CI limits were < 1 in 12 ICUs and upper RALOSR 95% CI limits were > 1 in 19; for the LMM these counts were 14 and 14 respectively.

##### OMELOS

**Fig. 5:**
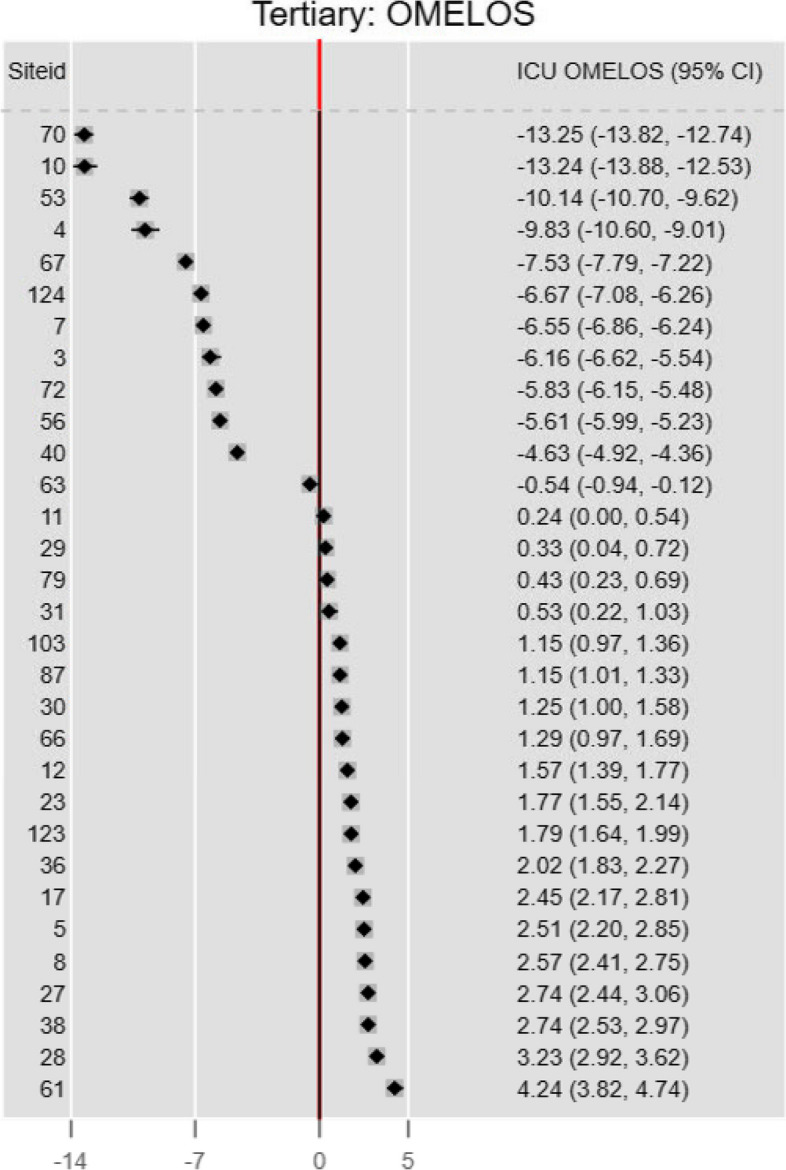
OMELOS (from GLMM)

The OMELOS fixed effects estimates are shown in Fig. 5. There was no concordance of ICU rankings compared with the RALOSR for either the GLMM or LMM models. The upper 95% CI limits were < 0 in 12 ICUs and lower 95% CI limits were > 0 in 19.

### Site-specific random effects

**Fig. 6:**
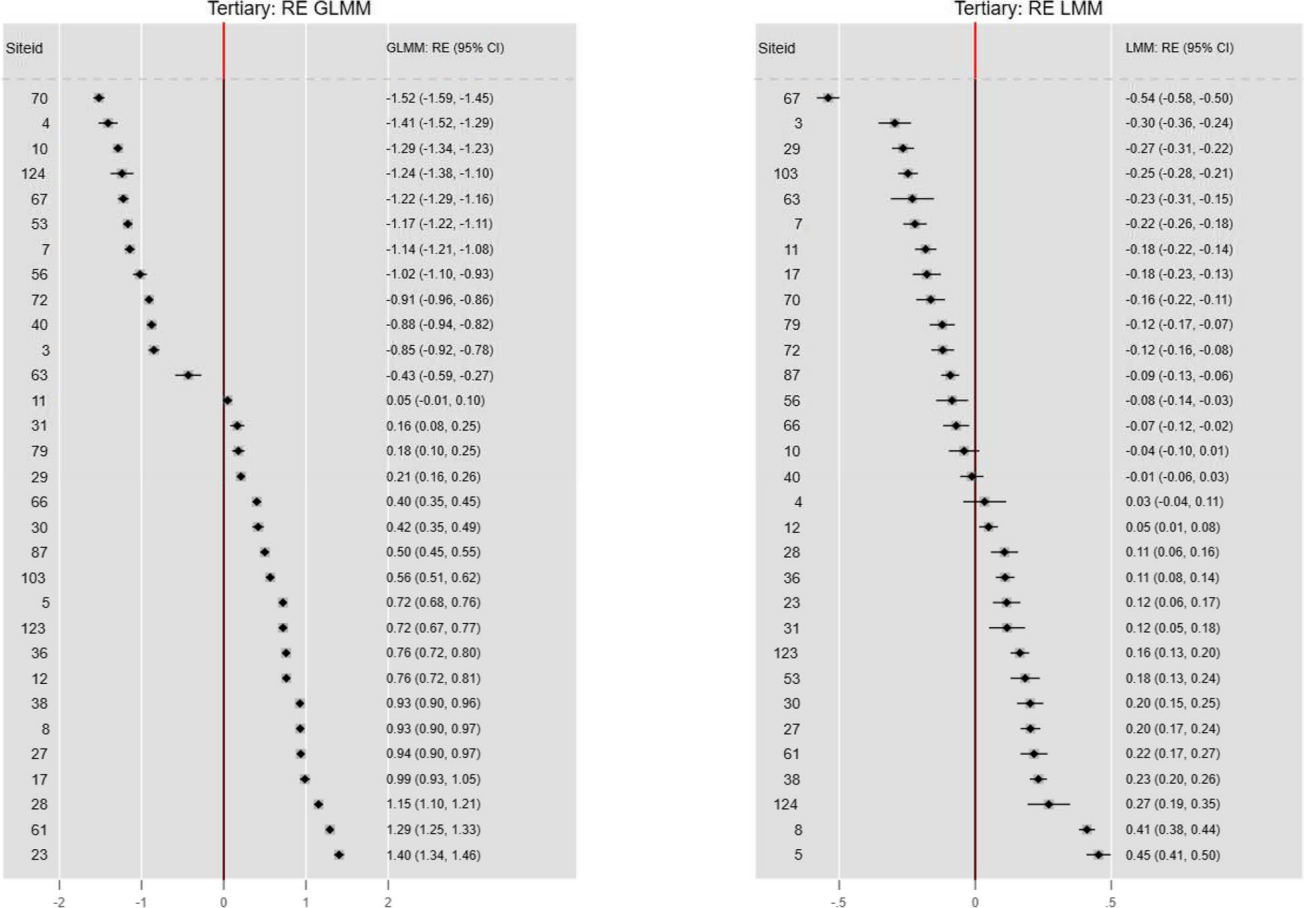
Site specific RE: GLMM and LMM

The ICU site RE are plotted in Fig. 6 for both the GLMM and LMM models. There was no concordance of ICU rankings between the two model RE and the LMM RE were constrained in magnitude compared with the GLMM. The GLM and LMM RE upper 95% CI limits were < 0 in 12 and 14 ICUs and lower 95% CI limits were > 0 in 18 and 14 respectively. Not surprisingly, ICU rankings were discordant between the RE and FE models.

### ICU site rankings

#### Marginal confidence sets: RALOSR: GLMM versus LMM

**Fig. 7:**
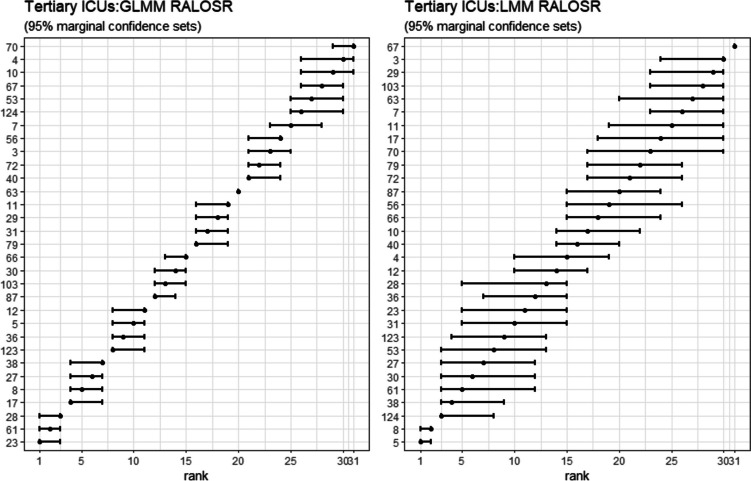
Marginal confidence sets for RALOSR: GLMM and LMM

Figure 7 shows marginal ICU site rankings estimated for the RALOSR for both GLMM and LMM (fixed effects) as estimated by the “csranks” package. The interpretation of “marginal” is that the confidence set covers a single ICU LOS (ranking point estimate) with probability 95%.

For marginal confidence sets, the GLMM produces clusters of similarly ranked ICUs, but for the LMM the rankings were far more concentrated and the 95% limits are wider.

#### Simultaneous confidence sets: RALOSR: GLMM versus LMM

**Fig. 8:**
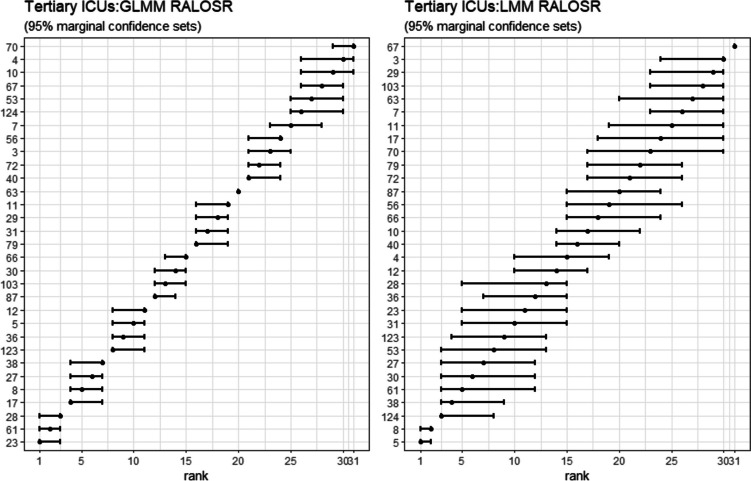
Simultaneous confidence sets for RALOSR, GLMM and LMM

Figure 8 shows simultaneous ICU site rankings estimated for the RALOSR for both GLMM and LMM (fixed effects). The interpretation of “simultaneous” is that the confidence sets simultaneously cover all differences in ICU RALOSR with 95% probability. Site rank clustering for GLMM is less apparent than for the marginal sets and the simultaneous confidence sets are more concentrated than in the marginal case. Simultaneous 95% limits were wider for both estimators.

#### OMELOS from GLMM

**Fig. 9:**
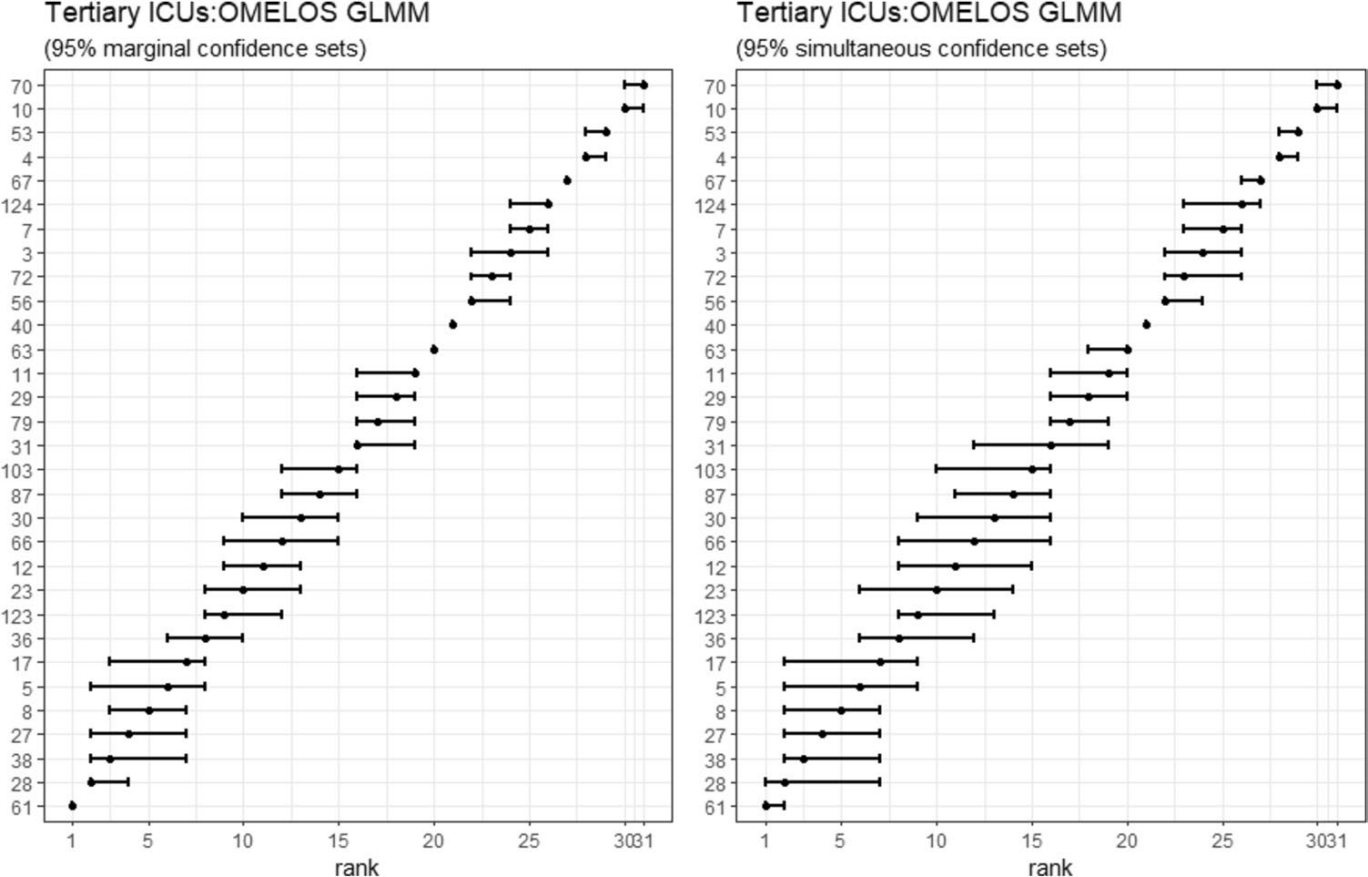
Marginal and simultaneous confidence sets for the OMELOS metric

Figure 9 shows marginal and simultaneous confidence sets for the OMELOS metric (fixed effects). Marginal rank clustering appears less marked than for RALOSR, both GLMM and LMM. Simultaneous set ranking still preserved some clustering features; 95% confidence limits were wider.

#### Site-specific RE: GLMM and LMM

**Fig. 10:**
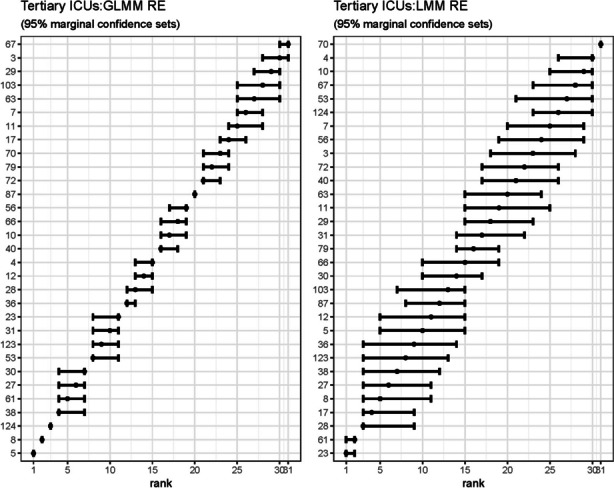
Marginal confidence sets for ICU RE ranks: GLMM and LMM

Figure 10 shows the marginal confidence sets for the ICU site-specific ranked RE for both the GLMM and the LMM. The GLMM shows clustering of the site RE, whereas the LMM estimates are compressed, with wider 95% limits.

**Fig. 11:**
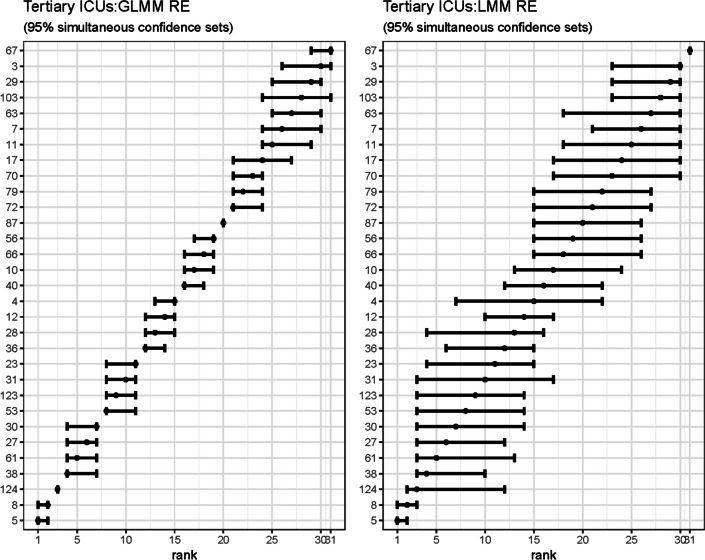
Simultaneous confidence sets for ICU RE ranks: GLMM and LMM

Figure 11 shows the simultaneous confidence sets for ranked ICU RE for both the GLMM and the LMM. The GLMM shows clustering of the site RE, whereas the LMM estimates are compacted, with wider 95% limits.

## Discussion

Both the GLMM and LMM performed satisfactorily with respect to model specification and prediction of ICU LOS. However, there was no concordance of ICU rankings between model predictions, GLMM versus LMM, nor for the quality metrics used, RALOSR, OMELOS and site-specific RE. That is, there was no “one best model”; thus, ICU “performance” is determined by model choice and any rankings thereupon should be circumspect. These inconsistencies are further examined.

### Predictive models

Within the critical care literature prediction of ICU LOS has predominately used linear regression [8, 31], generalised linear regression (GLM [32, 33]) and LMM [6, 10], the latter formally accounting for patient clustering within ICUs. Although GLM variants, (Poisson, negative binomial and Gamma) including the mixed model (RE) formulation [33, 34] have also been utilised, the current study, despite detailed examination, found lack of convergence with mixed effects Poisson and gamma models, possibly related to the large cohort size and multiple factor interactions. In the current study, the maximum ICU LOS was 127 days and there were no negative predicted LOS days, as may occur with linear regression with raw ICU LOS [33]. No formal truncation of LOS was undertaken; the implications of these measures have been previously discussed in detail [6].

The *R*^2^ for both models at the patient and ICU level for predicted LOS were reasonable. At the patient and ICU level *R*^2^ values of 20–28% and 50–70% respectively have been found for predictive models [5, 7]. This being said, the current study operated at the ICU level and a focus on performance at the individual level would not seem to be warranted nor an intrinsically productive exercise [7, 8]. For other right skewed variables, such as health costs, there would appear to be an upper limitation to *R*^2^ [35] and comparison of *R*^2^ values between models with different functional forms of the dependent variable, for instance raw and log transformed [31, 33], is not a justifiable practice [36]. Formal computational *R*^2^ measures have been described for both LMM and GLMM [37, 38], but a simple easily computed measure was preferred. Uncertainty, as confidence intervals (CI), has been variously estimated; analytic [39] or by the bootstrap [40], of which there are a “bewildering” array of methods [41].

LOS, either ICU or hospital, is positively right skewed and log transformation has been frequently applied to LOS as the dependent regression variable. This being said, appropriate retransformation [42, 43] to the original metric (days) is problematical as $\mathrm{exp}\left\{E\left(\mathrm{ln\, }y\right)\right\}\ne E\left(Y\right)$ and has rarely been addressed within the biomedical as opposed to the econometric [6, 44] literature. Although correction terms for back transformation to the original metric under both homo- and hetero-skedasticity have been implemented in Stata for linear regression models [45], such is not the case for LMM, albeit the theoretical basis for such has been established by Ramierez-Aldana and Naranjo [46].

There has been debate regarding the virtues, or otherwise, of log transformation in analysis [16]. In (linear) regression, the requirement for “normality” applies to model residuals not to the data covariates [47] and log transformation guarantees neither reduced dependent variable skewness nor variation; in fact, it may produce the opposite [48]. In the current case, normality of ICU LOS was not attained by log transformation, implying that the raw ICU LOS was not log-normally distributed. With respect to inference on the additive (arithmetic [6]) or multiplicative (geometric [10]) scale, the geometric mean, being multiplicative, has found use in analysing compounding investment [49] and the physical sciences [50], but lacks a “clear and concise physical interpretation” [51]. It exhibits bias for small samples and is sensitive to the probability distribution and skewness of the variable under consideration; only for the lognormal distribution is the geometric mean equivalent to the median [51]. For skewed data sets with many zeros, the common practice of adding a small positive constant to the observations (the “shift” parameter) before log transformation has little to recommend it as such a parameter has a highly significant effect on the estimator of the geometric mean [16]. Recent reviews have cautioned against the “routine” use of log-transformation in regression; rather GLM, or, as in the current paper, GLMM have been endorsed [14, 52, 53]. As noted by Deb et al., “Properly interpreting results from a log-transformed model requires substantially more effort” [54].

### Quality measures

ICU LOS would seem to be an exemplary quality measure, for reflecting resource use [3] and has been used with outcome measures, such as the standardised mortality rate (SMR), in “efficiency plots” [2] in a number of jurisdictions [39, 55–57]. Empirical studies have also demonstrated independence of indices of ICU LOS and the SMR [9, 31, 58].

Using three ICU LOS indices, OMELOS, RALOSR and site-specific RE, with two estimators of ICU LOS (GLMM and LMM), there was no monotonicity of ICU LOS point-estimate nor rankings between indices and or estimator. No intrinsic merit of one or more of these indices / estimators would appear to have been demonstrated, although attention has been drawn to potential limitations of the geometric (mean) metric and it could be argued that, ceteris paribus, site-specific RE encapsulate ICU differences more adroitly [59]. Caterpillar plots have been used to display indices of RALOS [9, 10], but the debate regarding the appropriate way to analyse and present such data, since the seminal paper (1996) of Goldstein and Healy, “The graphical presentation of a collection of means” [60], is substantial [59, 61]. One particular problem with the caterpillar and forest plot [62] variant is that of “…eyeballing …” the estimates, whereby inference (of, say, ICU differences) is conducted in a non-transparent manner [63]. Formal solutions to this problem have been proposed [21, 64], but the current study used ranking measures. Rankings are estimates, not true values, and such uncertainty may be addressed by constructing confidence sets for the ICU LOS ranks as (i) marginal, the confidence set covers a single ICU LOS with 95% probability and (ii) simultaneous, the confidence sets simultaneously cover all differences in ICU LOS with 95% probability [12]. As implemented in the “csranks” software [29], the multiple hypothesis testing regimen controls the familywise error rate and any false directional claim about the sign of a difference; the assumptions involved are “weak” and robust to small differences between (ICU) units ([12], especially “Remark 3.5”). Not surprisingly, the ranking estimates and conventional point-estimates and 95% CI across quality indices and estimator were not consistent, but the former more easily displayed ICU clustering (small measure estimate differences) and simultaneous inference across ICUs. Ranking estimates for all hospital ICU classifications and quality metrics are displayed in Supplementary files: Appendix II. With respect to between-ICU discrimination, the OMELOS metric would appear to be most favourable for both marginal and simultaneous confidence sets, although this was not as explicit in the rural / regional ICU cohort. This may reflect practice patterns within ICU cohorts and / or ICU patient yearly number; the latter varied substantially over ICU hospital classification (Table 1), as expected. We view the utilisation of the confidence sets for the ICU LOS ranks as a major advancement.

### Implications of the current study

The upshot of our analysis is that there is no “one best model”; each model produced different rankings. ICUs may be unfairly labelled as “poor performers” when using a particular risk-adjustment model and deemed “good performers” when using a different model. “Performance” in this context may represent quality of care or stewardship of limited resources. Casting a hospital as a “poor performer” may not only negatively affect their reimbursement but may also negatively impact their standing in the community. As such, a multifarious approach to the development and testing of future predictive and risk-adjustment models is mandated to ensure that only the “one best model” is promulgated. Conversely, if multiple models produce different rankings (as we found here), then no one model should be proffered as the definitive solution for risk-adjustment.

### Limitations

The current study was registry derived [20] and it is known that clinical studies using observational databases may be sensitive to database choice [65]. Only two estimators of LOS have been reported, albeit many potential estimators exist; the performance of some of these have been discussed in detail [6]. Death in ICU was also treated as a fixed model covariate rather than censored, as in time-to-event analysis, to facilitate straightforward analysis of total ICU population. Similarly, ICU LOS was analysed as a quality-of-care indicator and not hospital LOS, as the former appears to be the most plausible choice, at least within the critical care literature; more particularly in so-called “efficiency plots”. The models entailed a large number of associated covariates, but the “problem” of covariate multicollinearity was discounted [66]. The impact of “exit block” upon ICU LOS [67] was not subject to quantification.

## Conclusions

Inference regarding adjusted ICU LOS was dependent upon the statistical estimator and the quality index used to quantify any LOS differences. Therefore, formal ranking estimates, being subject to model determination, are problematic. Development and testing of future predictive and risk-adjustment models should utilize a comprehensive approach, such as that implemented here, to test the consistency of different models in producing ICU rankings.

### Supplementary Information

**Additional file 1.**

## Data Availability

The dataset is the property of the ANZICS CORE and contributing ICUs and is not in the public domain. Access to the data by researchers, submitting ICUs, jurisdictional funding bodies and other interested parties is obtained under specific conditions and upon written request (“ANZICS CORE Data Access and Publication Policy.pdf”, http://www.anzics.com.au/Downloads/ANZICS%20CORE%20Data%20Access%20and%20Publication%20Policy%20July%202017.pdf).

## References

[1] Becker RB, Zimmerman JE, Knaus WA, Wagner DP, Seneff MG, Draper EA, Higgins TL, Estafanous FG, Loop FD (1995). The use of APACHE III to evaluate ICU length of stay, resource use, and mortality after coronary artery by-pass surgery. J Cardiovasc Surg.

[2] Rapoport J, Teres D, Lemeshow S, Gehlbach S (1994). A method for assessing the clinical-performance and cost-effectiveness of intensive-care units - a multicenter inception cohort study. Crit Care Med.

[3] Rapoport J, Teres D, Zhao Y, Lemeshow S (2003). Length of stay data as a guide to hospital economic performance for ICU patients. Med Care.

[4] Peres IT, Hamacher S, Cyrino Oliveira FL, Tavares Thome AM, Bozza FA (2020). What factors predict length of stay in the intensive care unit? Systematic review and meta-analysis. J Crit Care.

[5] Verburg IWM, Atashi A, Eslami S, Holman R, Abu-Hanna A, de Jonge E, Peek N, de Keizer NF (2017). Which models can I use to predict adult ICU length of stay? A systematic review. Crit Care Med.

[6] Moran J, Solomon P (2012). A review of statistical estimators for risk-adjusted length of stay: analysis of the Australian and new Zealand intensive care adult patient data-base, 2008–2009. BMC Med Res Methodol.

[7] Kramer AA (2017). Are ICU length of stay predictions worthwhile?. Crit Care Med.

[8] Zimmerman JE, Kramer AA, McNair DS, Malila FM, Shaffer VL (2006). Intensive care unit length of stay: benchmarking based on Acute Physiology and Chronic Health Evaluation (APACHE) IV*. Crit Care Med.

[9] Render ML, Kim HM, Deddens J, Sivaganesin S, Welsh DE, Bickel K, Freyberg R, Timmons S, Johnston J, Connors AF (2005). Variation in outcomes in Veterans Affairs intensive care units with a computerized severity measure. Crit Care Med.

[10] Straney LD, Udy AA, Burrell A, Bergmeir C, Huckson S, Cooper DJ, Pilcher DV (2017). Modelling risk-adjusted variation in length of stay among Australian and New Zealand ICUs. PLoS One.

[11] Hurley JC (2020). Forrest plots or caterpillar plots?. J Clin Epidemiol.

[12] Mogstad M, Romano JP, Shaikh AM, Wilhelm D. Inference for ranks with applications to mobility across neighborhoods and academic achievement across countries. 2022. Available @ https://home.uchicago.edu/~amshaikh/webfiles/rankingsconf.pdf; Downloaded: 6th June 2022.

[13] Little RJ, Lewis RJ (2021). Estimands, estimators, and estimates. JAMA.

[14] Lo S, Andrews S (2015). To transform or not to transform: using generalized linear mixed models to analyse reaction time data. Front Psychol.

[15] Becker TE, Robertson MM, Vandenberg RJ (2019). Nonlinear transformations in organizational research: possible problems and potential solutions. Organ Res Methods.

[16] Feng C, Wang H, Lu N, Tu XM (2013). Log transformation: application and interpretation in biomedical research. Stat Med.

[17] Feng C, Wang H, Lu N, Chen T, He H, Lu Y, Tu XM (2014). Log-transformation and its implications for data analysis. Shanghai Arch Psychiatry.

[18] Curto JD. Inference about the arithmetic average of log transformed data. Statistical Papers; 2022. Available @ https://link.springer.com/article/101007/s00362-022-01315-x.

[19] ANZICS_CORE. ANZICS CORE data access and publication policy. 2017. Available @ https://www.anzics.com.au/data-access-and-publication-policy/. Downloaded 5th November 2022.

[20] Stow PJ, Hart GK, Higlett T, George C, Herkes R, McWilliam D, Bellomo R (2006). Development and implementation of a high-quality clinical database: the Australian and New Zealand Intensive Care Society Adult Patient Database. J Crit Care.

[21] Moran JL, Solomon PJ, ANZICS-CORE (2014). Fixed effects modelling for provider mortality outcomes: analysis of the Australia and New Zealand Intensive Care Society (ANZICS) adult patient data-base. PLoS One.

[22] Breiman L (2001). Statistical modeling: the two cultures. Stat Sci.

[23] Barber JA, Thompson SG (2000). Analysis of cost data in randomized trials: an application of the non-parametric bootstrap. Stat Med.

[24] Cox NJ (2007). Kernel estimation as a basic tool for geomorphological data analysis. Earth Surf Proc Land.

[25] Alejo J, Bera A, Montes-Rojas G, Galvao A, Xiao Z (2016). Tests for normality based on the quantile-mean covariance. Stand Genomic Sci.

[26] StataCorp. Stata multilevel mixed effects reference manual release 17. 2021. pp. 127–128, 552–554. Available @ https://www.stata.com/manuals/me.pdf; downloaded 21st January 2022.

[27] StataCorp. estatic—display information criteria (version 18). 2023. Available @ https://www.stata.com/manuals/restatic.pdf.

[28] Fisher D. metan: module for fixed and random effects meta-analysis. Available @ http://fmwww.bc.edu/RePEc/bocode/m; Downloaded December 10th 2021.

[29] Wilhelm D. csranks: R package for confidence sets for ranks. Available @ https://danielwilhelm.github.io/R-CS-ranks/; downloaded 12th January 2022.

[30] Harhay MO, Ratcliffe SJ, Small DS, Suttner LH, Crowther MJ, Halpern SD (2019). Measuring and analyzing length of stay in critical care trials. Med Care.

[31] Niskanen M, Reinikainen M, Pettilä V (2009). Case-mix-adjusted length of stay and mortality in 23 Finnish ICUs. Intensive Care Med.

[32] Moran JL, Solomon PJ, Peisach AR, Martin J (2007). New models for old questions: Generalized Linear Models for cost prediction. J Eval Clin Pract.

[33] Verburg IWM, de Keizer NF, de Jonge E, Peek N (2014). Comparison of regression methods for modeling intensive care length of stay. PLoS One.

[34] Straney L, Clements A, Alexander J, Slater A, Group ftAPS (2010). Quantifying variation of paediatric length of stay among intensive care units in Australia and New Zealand. Qual Saf Health Care.

[35] Diehr P, Yanez D, Ash A, Hornbrook M, Lin DY (1999). Methods for analyzing health care utilization and costs. Annu Rev Public Health.

[36] Wooldridge JM. Multiple regression analytics: further issues. In: Introductory econometrics: a modern approach. 5th edn. Mason: South-Western; 2013. pp. 204–205.

[37] Rights JD, Sterba SK (2020). New recommendations on the use of R-squared differences in multilevel model comparisons. Multivar Behav Res.

[38] Stoffel MA, Nakagawa S, Schielzeth H (2021). partR2: partitioning R-2 in generalized linear mixed models. Peerj.

[39] Straney LD, Clements A, Alexander J, Slater A, Grp APS (2010). Measuring efficiency in Australian and New Zealand paediatric intensive care units. Intensive Care Med.

[40] Nathanson BH, Higgins TL, Teres D, Copes WS, Kramer A, Stark M (2007). A revised method to assess intensive care unit clinical performance and resource utilization. Crit Care Med.

[41] Carpenter J, Bithell J (2000). Bootstrap confidence intervals: when, which, what? A practical guide for medical statisticians. Stat Med.

[42] Manning WG, Mullahy J (2001). Estimating log models: to transform or not to transform?. J Health Econ.

[43] Manning WG (1998). The logged dependent variable, heteroscedasticity, and the retransformation problem. J Health Econ.

[44] Duan N (1983). Smearing estimate: a nonparametric retransformation method. J Am Stat Assoc.

[45] Kranker K. predlog2 - Duan smearing & heteroscedastic smearing retransformation. 2019. Available @ https://github.com/kkranker/kk-adofiles/blob/master/README.md.

[46] Ramirez-Aldana R, Naranjo L (2021). Random intercept and linear mixed models including heteroscedasticity in a logarithmic scale: correction terms and prediction in the original scale. PLoS One.

[47] Buntin MB, Zaslavsky AM (2004). Too much ado about two-part models and transformation? Comparing methods of modeling Medicare expenditures. J Health Econ.

[48] Feng C, Wang H, Lu N, Tu XM (2013). Response to comments on ‘Log transformation: application and interpretation in biomedical research’. Stat Med.

[49] Jacquier E, Kane A, Marcus AJ (2003). Geometric or arithmetic mean: a reconsideration. Financ Anal J.

[50] Mahajan S (2019). Don’t demean the geometric mean. Am J Phys.

[51] Vogel RM (2022). The geometric mean?. Commun Stat Theory Methods.

[52] Ronkko M, Aalto E, Tenhunen H, Aguirre-Urreta MI (2022). Eight simple guidelines for improved understanding of transformations and nonlinear effects. Organ Res Methods.

[53] Villadsen AR, Wulff JN (2021). Statistical myths about log-transformed dependent variables and how to better estimate exponential models. Br J Manag.

[54] Deb P, Norton EC, Manning WG. Log and box-cox models. In: Health econometrics using Stata. College Station: Stata Press; 2017. p. 93–103.

[55] Rothen HU, Takala J (2008). Can outcome prediction data change patient outcomes and organizational outcomes?. Curr Opin Crit Care.

[56] Takala J, Moser A, Raj R, Pettila V, Irincheeva I, Selander T, Kiiski O, Varpula T, Reinikainen M, Jakob SM (2022). Variation in severity-adjusted resource use and outcome in intensive care units. Intensive Care Med.

[57] Burrell AJC, Udy A, Straney L, Huckson S, Chavan S, Saethern J, Pilcher D (2021). “The ICU efficiency plot”: a novel graphical measure of ICU performance in Australia and New Zealand. Crit Care Resusc.

[58] Dominguez L, Enriquez P, Alvarez P, de Frutos M, Sagredo V, Dominguez A, Collado J, Taboada F, Garcia-Labattut A, Bobillo F (2008). Mortality and hospital stay adjusted for severity as indicators of effectiveness and efficiency of attention to intensive care unit patients. Med Intensiva.

[59] Afshartous D, Wolf M (2007). Avoiding ‘data snooping’ in multilevel and mixed effects models. J R Stat Soc Ser A Stat Soc.

[60] Goldstein H, Healy MJR (1995). The graphical presentation of a collection of means. J R Stat Soc A.

[61] Goldstein H. Using league table rankings in public policy formation: statistical issues. In: Fienberg SE, editor. Annual review of statistics and its application, Vol 1. 2014. pp. 385–399.

[62] Li G, Zeng J, Tian J, Levine MAH, Thabane L (2020). Multiple uses of forest plots in presenting analysis results in health research: a tutorial. J Clin Epidemiol.

[63] Röver C, Friede T (2020). Dynamically borrowing strength from another study through shrinkage estimation. Stat Methods Med Res.

[64] Afshartous D, Preston RA (2010). Confidence intervals for dependent data: equating non-overlap with statistical significance. Comput Stat Data Anal.

[65] Madigan D, Ryan PB, Schuemie M, Stang PE, Overhage JM, Hartzema AG, Suchard MA, DuMouchel W, Berlin JA (2013). Evaluating the impact of database heterogeneity on observational study results. Am J Epidemiol.

[66] Lindner T, Puck J, Verbeke A (2020). Misconceptions about multicollinearity in international business research: identification, consequences, and remedies. J Int Bus Stud.

[67] Anstey MH, Thompson K, Seppelt I (2017). Exit block in the intensive care unit. Med J Aust.

