# Supplementary material for: Modelling of intensive care unit (ICU) length of stay as a quality measure: a problematic exercise
Source: BMC Med Res Methodol. 2023 Sep 14;23:207. doi: 10.1186/s12874-023-02028-x (PMC10500937; doi:10.1186/s12874-023-02028-x)
Supplement: Supplementary file 1 — Additional file 1. [file 12874_2023_2028_MOESM1_ESM.docx]

**Appendix I**

**Table 1.**

|  | **Total** |
| --- | --- |
|  | **N=94,361** |
| Collapsed APACHE III diagnostic categories |  |
| Cardiovascular_medical | 7.5% (7,071) |
| Respiratory medical | 9.6% (9,063) |
| Liver_GIS_medical | 2.5% (2,398) |
| CNS_medical | 5.0% (4,763) |
| Sepsis | 6.7% (6,285) |
| Trauma | 2.9% (2,719) |
| Metabolic Hormonal | 6.3% (5,960) |
| Haematologic | 0.3% (317) |
| Renal_GUS | 1.4% (1,340) |
| Other medical disorders | 0.5% (459) |
| Musculoskeletal / Skin | 0.2% (227) |
| Cardio-Vascular surgery | 2.4% (2,238) |
| Thoracic surgery | 1.0% (942) |
| GIS surgery | 4.5% (4,277) |
| CNS surgery | 1.5% (1,395) |
| Traumatic/Orthopaedic surgery | 1.4% (1,286) |
| Renal_GUS surgery | 0.6% (561) |
| Gynaecological | 0.6% (610) |
| Musculoskeletal / Skin Surgery | 1.4% (1,330) |
| Haematologic Surgery | 0.0% (23) |
| Metabolic Surgery | 0.1% (101) |
| Cardiovascular surgery elective | 18.5% (17,476) |
| Thoracic surgery elective | 3.8% (3,617) |
| GIS surgery elective | 7.2% (6,751) |
| CNS surgery elective | 6.5% (6,094) |
| Traumatic/Orthopaedic surgery elective | 0.1% (135) |
| Renal_GUS surgery elective | 1.4% (1,349) |
| Gynaecological surgery elective | 0.9% (820) |
| Musculoskeletal / Skin Surgery elective | 4.4% (4,158) |
| Metabolic Surgery elective | 0.6% (571) |

Data are presented as % (n). GIS, gastro intestinal. CNS, central nervous system.

GUS, genito-urinary.

**Stata command syntax and model specification**

GLMM

**meglm icudays i.diedicu##(c.c_ap3score) c.c_ap3sqr ln_anzrod ///**

**c_age c_agesqr c_ap3score c_ap3sqr c.c_age##(i.acuterf) i.carrest ///**

**c.c_ap3score##( i.trlimited i.ventilated i.hoslevel) c.preicudys2 ///**

**i.ap3diagnosis2##c.c_ap3score ///**

**b1.i.annvol_deciles##(c.c_ap3score c.c_age) || siteid:, family(gau) link(log) ///**

**difficult technique(bfgs)**

1. **unary operator to specify indicators**

**c. unary operator to treat as continuous**

**## binary operator to specify factorial interactions**

**c_ centred**

**|| re_equation**

**difficult use a different stepping algorithm in nonconcave regions**

**bfgs specifies the Broyden-Fletcher-Goldfarb-Shanno (BFGS) algorithm.**

LMM

**mixed logicudays i.diedicu##(c.c_ap3score) c.c_ap3sqr ln_anzrod ///**

**c_age c_agesqr c_ap3score c_ap3sqr c.c_age##(i.acuterf) i.carrest ///**

**c.c_ap3score##( i.trlimited i.ventilated i.hoslevel) c.preicudys2 ///**

**i.ap3diagnosis2##c.c_ap3score ///**

**b1.i.annvol_deciles##(c.c_ap3score c.c_age) || siteid:**

Model covariate specification was undertaken using information criteria, using Akaike (AIC) and Schwartz’s Bayesian (BIC) criteria [1].

The question of the role of ICU death as a predictor of LOS is worth comment. We noted (Statistical analysis, P5, l15-17) that “The primary focus was the prediction of RALOS and not on coefficient interpretation, albeit subscribing to a data- not algorithmic-modeling scenario, as defined in Breiman 2001 [22]”. Thus, we include “diedicu” as a binary predictor in the model syntax and interact this predictor with a patient severity of illness predictor, the well validated APACHE III score [2]. Of note, both Angus et al [3] and Straney et al [4] (our reference 10) use “death” as a model predictor. We included a similar set of variables in our models, in an effort to compare consistency of results (ICU rankings) with existing modelling approaches.

For both the GLMM and LMM, the addition of the covariate “diedicu” improved model fit as adjudged by *both* AIC and BIC. This was also the result for the so-called “consistent” AIC (a consistent version of AIC; that is, the probability of selecting the "true model" approaches 1 as sample size increases [5]).

**Table 2. GLMM: model coefficients**

|  | Odds Ratio | p-value | 95% CI |
| --- | --- | --- | --- |
| ICU LOS |  |  |  |
| Died | 1.403 | 0.000 | 1.351 1.457 |
| APIII score | 1.005 | 0.000 | 1.003 1.007 |
| Died # APIII score | 0.982 | 0.000 | 0.981 0.983 |
| APIII score sqr. | 1.000 | 0.000 | 1.000 1.000 |
| Log ANZROD | 1.195 | 0.000 | 1.173 1.218 |
| Age | 0.991 | 0.000 | 0.989 0.993 |
| Age_sqr. | 1.000 | 0.000 | 1.000 1.000 |
| Acute renal failure | 1.278 | 0.000 | 1.245 1.311 |
| Acute renal failure # Age | 0.998 | 0.002 | 0.996 0.999 |
| Cardiac arrest | 0.869 | 0.000 | 0.834 0.907 |
| Treatment limitation | 0.726 | 0.000 | 0.684 0.770 |
| Ventilated | 1.623 | 0.000 | 1.586 1.662 |
| Private | 1.320 | 0.162 | 0.895 1.948 |
| Rural / Regional | 0.702 | 0.150 | 0.434 1.136 |
| Tertiary | 1.969 | 0.004 | 1.246 3.111 |
| Treatment limitation # APIII score | 1.000 | 0.784 | 0.998 1.001 |
| Ventilated # APIII score | 0.999 | 0.008 | 0.998 1.000 |
| Private # APIII score | 1.003 | 0.000 | 1.001 1.004 |
| Rural / Regional # APIII score | 1.000 | 0.889 | 0.999 1.001 |
| Tertiary # APIII score | 1.001 | 0.009 | 1.000 1.002 |
| Pre-ICU days | 1.001 | 0.245 | 0.999 1.004 |
| Respiratory medical | 1.313 | 0.000 | 1.257 1.372 |
| Liver_GIS_medical | 1.267 | 0.000 | 1.194 1.346 |
| CNS_medical | 1.157 | 0.000 | 1.103 1.214 |
| Sepsis | 1.206 | 0.000 | 1.148 1.268 |
| Trauma | 1.312 | 0.000 | 1.244 1.385 |
| Metabolic Hormonal | 0.734 | 0.000 | 0.681 0.792 |
| Haematologic | 1.058 | 0.420 | 0.923 1.212 |
| Renal_GUS | 0.761 | 0.000 | 0.677 0.855 |
| Other medical disorders | 0.878 | 0.105 | 0.750 1.028 |
| Musculoskeletal / Skin | 1.342 | 0.000 | 1.169 1.541 |
| Cardio-Vascular surgery | 1.034 | 0.343 | 0.965 1.106 |
| Thoracic surgery | 0.934 | 0.139 | 0.852 1.023 |
| GIS surgery | 0.903 | 0.001 | 0.850 0.959 |
| CNS surgery | 1.314 | 0.000 | 1.237 1.395 |
| Traumatic/Orthopaedic surgery | 1.583 | 0.000 | 1.494 1.676 |
| Renal_GUS surgery | 0.818 | 0.013 | 0.698 0.959 |
| Gynaecological | 0.512 | 0.000 | 0.404 0.649 |
| Musculoskeletal / Skin Surgery | 0.916 | 0.067 | 0.833 1.006 |
| Haematologic Surgery | 1.158 | 0.596 | 0.673 1.992 |
| Metabolic Surgery | 0.647 | 0.041 | 0.426 0.983 |
| Cardiovascular surgery elective | 0.813 | 0.000 | 0.769 0.860 |
| Thoracic surgery elective | 0.689 | 0.000 | 0.623 0.760 |
| GIS surgery elective | 0.907 | 0.002 | 0.854 0.965 |
| CNS surgery elective | 0.784 | 0.000 | 0.717 0.857 |
| Traumatic/Orthopaedic surgery elective | 0.910 | 0.545 | 0.669 1.237 |
| Renal_GUS surgery elective | 0.798 | 0.001 | 0.699 0.911 |
| Gynaecological surgery elective | 0.571 | 0.000 | 0.426 0.765 |
| Musculoskeletal / Skin Surgery elective | 0.712 | 0.000 | 0.645 0.785 |
| Metabolic Surgery elective | 0.458 | 0.001 | 0.293 0.717 |
| Respiratory medical # APIII score | 0.999 | 0.284 | 0.998 1.001 |
| Liver_GIS_medical # APIII score | 0.996 | 0.000 | 0.994 0.998 |
| CNS_medical # APIII score | 0.992 | 0.000 | 0.990 0.993 |
| Sepsis # APIII score | 1.002 | 0.003 | 1.001 1.003 |
| Trauma # APIII score | 0.999 | 0.150 | 0.997 1.000 |
| Metabolic Hormonal # APIII score | 1.000 | 0.788 | 0.998 1.002 |
| Haematologic # APIII score | 1.004 | 0.003 | 1.001 1.007 |
| Renal_GUS # APIII score | 1.005 | 0.002 | 1.002 1.007 |
| Other medical disorders # APIII score | 0.999 | 0.848 | 0.994 1.005 |
| Musculoskeletal / Skin # APIII score | 1.001 | 0.694 | 0.996 1.005 |
| Cardio-Vascular surgery # APIII score | 1.004 | 0.000 | 1.002 1.006 |
| Thoracic surgery # APIII score | 1.003 | 0.143 | 0.999 1.007 |
| GIS surgery # APIII score | 1.002 | 0.012 | 1.000 1.004 |
| CNS surgery # APIII score | 0.999 | 0.309 | 0.997 1.001 |
| Traumatic/Orthopaedic surgery # APIII score | 0.999 | 0.306 | 0.997 1.001 |
| Renal_GUS surgery # APIII score | 1.005 | 0.016 | 1.001 1.009 |
| Gynaecological # APIII score | 0.999 | 0.913 | 0.988 1.011 |
| Musculoskeletal / Skin Surgery # APIII score | 1.003 | 0.011 | 1.001 1.006 |
| Haematologic Surgery # APIII score | 1.000 | 0.997 | 0.977 1.023 |
| Metabolic Surgery # APIII score | 1.013 | 0.088 | 0.998 1.029 |
| Cardiovascular surgery elective # APIII score | 1.005 | 0.000 | 1.004 1.007 |
| Thoracic surgery elective # APIII score | 0.999 | 0.831 | 0.995 1.004 |
| GIS surgery elective # APIII score | 1.002 | 0.155 | 0.999 1.004 |
| CNS surgery elective # APIII score | 1.004 | 0.045 | 1.000 1.008 |
| Traumatic/Orthopaedic surgery elective # APIII score | 0.999 | 0.850 | 0.988 1.010 |
| Renal_GUS surgery elective # APIII score | 0.999 | 0.862 | 0.992 1.007 |
| Gynaecological surgery elective # APIII score | 0.995 | 0.491 | 0.981 1.009 |
| Musculoskeletal / Skin Surgery elective # APIII score | 1.006 | 0.017 | 1.001 1.011 |
| Metabolic Surgery elective # APIII score | 0.991 | 0.409 | 0.970 1.012 |
| Deciles of annual patient volume  Decile 2 | 1.478 | 0.001 | 1.168 1.872 |
| Decile 3 | 0.289 | 0.000 | 0.227 0.367 |
| Decile 4 | 0.884 | 0.421 | 0.654 1.194 |
| Decile 5 | 1.574 | 0.007 | 1.130 2.194 |
| Decile 6 | 0.123 | 0.000 | 0.087 0.174 |
| Decile 7 | 0.267 | 0.000 | 0.187 0.383 |
| Decile 8 | 0.899 | 0.580 | 0.618 1.310 |
| Decile 9 | 0.320 | 0.000 | 0.219 0.467 |
| Decile 10 | 0.174 | 0.000 | 0.117 0.261 |
| Decile 2 # APIII score | 1.000 | 0.977 | 0.998 1.002 |
| Decile 3 # APIII score | 1.002 | 0.034 | 1.000 1.003 |
| Decile 4 # APIII score | 0.999 | 0.534 | 0.998 1.001 |
| Decile 5 # APIII score | 1.000 | 0.726 | 0.999 1.002 |
| Decile 6 # APIII score | 0.999 | 0.325 | 0.997 1.001 |
| Decile 7 # APIII score | 1.001 | 0.262 | 0.999 1.003 |
| Decile 8 # APIII score | 1.001 | 0.250 | 0.999 1.003 |
| Decile 9 # APIII score | 1.005 | 0.000 | 1.003 1.007 |
| Decile 10 # APIII score | 1.002 | 0.054 | 1.000 1.003 |
| Decile 2 # Age | 1.005 | 0.000 | 1.002 1.007 |
| Decile 3 # Age | 1.003 | 0.033 | 1.000 1.005 |
| Decile 4 # Age | 1.010 | 0.000 | 1.007 1.013 |
| Decile 5 # Age | 1.001 | 0.550 | 0.998 1.003 |
| Decile 6 # Age | 1.002 | 0.166 | 0.999 1.004 |
| Decile 7 # Age | 0.999 | 0.411 | 0.997 1.001 |
| Decile 8 # Age | 0.996 | 0.004 | 0.994 0.999 |
| Decile 9 # Age | 0.993 | 0.000 | 0.990 0.996 |
| Decile 10 # Age | 1.000 | 0.788 | 0.998 1.002 |
| Intercept | 6.041 | 0.000 | 4.203 8.682 |
|  |  |  |  |
| var(_cons[newsite]) | 0.698 |  | 0.525 0.930 |
| var(e.icudays) | 19.032 |  | 18.854 19.211 |

**Table 3: LMM: model coefficients**

|  | Coefficient | p-value | 95% CI |
| --- | --- | --- | --- |
| Log ICU LOS |  |  |  |
| Died | 0.096 | 0.000 | 0.056 0.136 |
| APIII score | 0.009 | 0.000 | 0.008 0.011 |
| Died # APIII score | -0.018 | 0.000 | -0.019 -0.017 |
| APIII score sqr. | -0.000 | 0.000 | -0.000 -0.000 |
| Log ANZROD | 0.124 | 0.000 | 0.113 0.134 |
| Age | -0.002 | 0.001 | -0.004 -0.001 |
| Age_sqr. | -0.000 | 0.000 | -0.000 -0.000 |
| Acute renal failure # Age | -0.001 | 0.433 | -0.002 0.001 |
| Cardiac arrest | -0.193 | 0.000 | -0.230 -0.155 |
| Treatment limitation | -0.175 | 0.000 | -0.206 -0.143 |
| Ventilated | 0.436 | 0.000 | 0.422 0.451 |
| Private | 0.030 | 0.551 | -0.069 0.129 |
| Rural / Regional | -0.017 | 0.784 | -0.139 0.105 |
| Tertiary | -0.044 | 0.460 | -0.162 0.073 |
| Treatment limitation # APIII score | -0.004 | 0.000 | -0.005 -0.003 |
| Ventilated # APIII score | -0.000 | 0.343 | -0.001 0.000 |
| Private # APIII score | 0.000 | 0.469 | -0.001 0.001 |
| Rural / Regional # APIII score | -0.002 | 0.000 | -0.003 -0.001 |
| Tertiary # APIII score | 0.000 | 0.316 | -0.000 0.001 |
| Pre-ICU days | 0.003 | 0.004 | 0.001 0.005 |
| Respiratory medical | 0.279 | 0.000 | 0.249 0.308 |
| Liver_GIS_medical | 0.173 | 0.000 | 0.131 0.216 |
| CNS_medical | 0.045 | 0.009 | 0.011 0.079 |
| Sepsis | 0.259 | 0.000 | 0.225 0.292 |
| Trauma | 0.274 | 0.000 | 0.234 0.314 |
| Metabolic Hormonal | -0.102 | 0.000 | -0.138 -0.066 |
| Haematologic | -0.030 | 0.583 | -0.136 0.077 |
| Renal_GUS | -0.013 | 0.634 | -0.067 0.041 |
| Other medical disorders | -0.135 | 0.002 | -0.220 -0.050 |
| Musculoskeletal / Skin | 0.277 | 0.000 | 0.168 0.385 |
| Cardio-Vascular surgery | 0.107 | 0.000 | 0.063 0.152 |
| Thoracic surgery | 0.022 | 0.473 | -0.038 0.082 |
| GIS surgery | -0.019 | 0.301 | -0.054 0.017 |
| CNS surgery | 0.219 | 0.000 | 0.170 0.269 |
| Traumatic/Orthopaedic surgery | 0.425 | 0.000 | 0.373 0.478 |
| Renal_GUS surgery | -0.032 | 0.398 | -0.105 0.042 |
| Gynaecological | -0.397 | 0.000 | -0.495 -0.299 |
| Musculoskeletal / Skin Surgery | -0.092 | 0.000 | -0.143 -0.042 |
| Haematologic Surgery | -0.032 | 0.861 | -0.394 0.329 |
| Metabolic Surgery | -0.298 | 0.004 | -0.498 -0.098 |
| Cardiovascular surgery elective | -0.011 | 0.505 | -0.044 0.022 |
| Thoracic surgery elective | -0.290 | 0.000 | -0.338 -0.242 |
| GIS surgery elective | -0.050 | 0.005 | -0.085 -0.015 |
| CNS surgery elective | -0.328 | 0.000 | -0.374 -0.282 |
| Traumatic/Orthopaedic surgery elective | -0.076 | 0.332 | -0.228 0.077 |
| Renal_GUS surgery elective | -0.103 | 0.001 | -0.163 -0.043 |
| Gynaecological surgery elective | -0.346 | 0.000 | -0.439 -0.253 |
| Musculoskeletal / Skin Surgery elective | -0.282 | 0.000 | -0.324 -0.239 |
| Metabolic Surgery elective | -0.469 | 0.000 | -0.591 -0.347 |
| Respiratory medical # APIII score | -0.002 | 0.001 | -0.002 -0.001 |
| Liver_GIS_medical # APIII score | -0.002 | 0.002 | -0.003 -0.001 |
| CNS_medical # APIII score | -0.008 | 0.000 | -0.009 -0.007 |
| Sepsis # APIII score | -0.000 | 0.862 | -0.001 0.001 |
| Trauma # APIII score | -0.003 | 0.000 | -0.005 -0.002 |
| Metabolic Hormonal # APIII score | -0.006 | 0.000 | -0.007 -0.005 |
| Haematologic # APIII score | 0.004 | 0.009 | 0.001 0.007 |
| Renal_GUS # APIII score | 0.003 | 0.001 | 0.001 0.005 |
| Other medical disorders # APIII score | 0.002 | 0.208 | -0.001 0.005 |
| Musculoskeletal / Skin # APIII score | 0.001 | 0.814 | -0.004 0.005 |
| Cardio-Vascular surgery # APIII score | 0.001 | 0.107 | -0.000 0.003 |
| Thoracic surgery # APIII score | 0.001 | 0.583 | -0.002 0.003 |
| GIS surgery # APIII score | 0.000 | 0.427 | -0.001 0.002 |
| CNS surgery # APIII score | -0.000 | 0.855 | -0.002 0.002 |
| Traumatic/Orthopaedic surgery # APIII score | -0.004 | 0.000 | -0.006 -0.003 |
| Renal_GUS surgery # APIII score | 0.001 | 0.429 | -0.002 0.004 |
| Gynaecological # APIII score | -0.007 | 0.000 | -0.011 -0.003 |
| Musculoskeletal / Skin Surgery # APIII score | 0.002 | 0.061 | -0.000 0.004 |
| Haematologic Surgery # APIII score | -0.012 | 0.145 | -0.028 0.004 |
| Metabolic Surgery # APIII score | -0.003 | 0.527 | -0.012 0.006 |
| Cardiovascular surgery elective # APIII score | -0.003 | 0.000 | -0.004 -0.002 |
| Thoracic surgery elective # APIII score | -0.006 | 0.000 | -0.008 -0.004 |
| GIS surgery elective # APIII score | -0.002 | 0.010 | -0.003 -0.000 |
| CNS surgery elective # APIII score | -0.009 | 0.000 | -0.011 -0.007 |
| Traumatic/Orthopaedic surgery elective # APIII score | -0.004 | 0.194 | -0.010 0.002 |
| Renal_GUS surgery elective # APIII score | -0.002 | 0.314 | -0.005 0.002 |
| Gynaecological surgery elective # APIII score | -0.008 | 0.000 | -0.012 -0.004 |
| Musculoskeletal / Skin Surgery elective # APIII score | -0.005 | 0.000 | -0.007 -0.003 |
| Metabolic Surgery elective # APIII score | -0.011 | 0.000 | -0.016 -0.005 |
| Deciles of annual patient volume  Decile 2 | -0.067 | 0.225 | -0.176 0.041 |
| Decile 3 | -0.182 | 0.002 | -0.301 -0.064 |
| Decile 4 | 0.014 | 0.863 | -0.145 0.173 |
| Decile 5 | -0.008 | 0.921 | -0.160 0.144 |
| Decile 6 | -0.039 | 0.595 | -0.181 0.104 |
| Decile 7 | -0.130 | 0.077 | -0.275 0.014 |
| Decile 8 | 0.038 | 0.645 | -0.124 0.200 |
| Decile 9 | -0.068 | 0.521 | -0.274 0.139 |
| Decile 10 | -0.181 | 0.022 | -0.335 -0.026 |
| Decile 2 # APIII score | 0.000 | 0.689 | -0.001 0.001 |
| Decile 3 # APIII score | 0.002 | 0.005 | 0.001 0.003 |
| Decile 4 # APIII score | 0.001 | 0.268 | -0.001 0.002 |
| Decile 5 # APIII score | -0.000 | 0.796 | -0.001 0.001 |
| Decile 6 # APIII score | -0.001 | 0.379 | -0.002 0.001 |
| Decile 7 # APIII score | 0.001 | 0.050 | -0.000 0.003 |
| Decile 8 # APIII score | 0.002 | 0.012 | 0.000 0.003 |
| Decile 9 # APIII score | 0.008 | 0.000 | 0.006 0.010 |
| Decile 10 # APIII score | 0.003 | 0.000 | 0.002 0.004 |
| Decile 2 # Age | -0.001 | 0.469 | -0.002 0.001 |
| Decile 3 # Age | -0.002 | 0.019 | -0.004 -0.000 |
| Decile 4 # Age | -0.000 | 0.740 | -0.002 0.002 |
| Decile 5 # Age | -0.002 | 0.057 | -0.003 0.000 |
| Decile 6 # Age | -0.001 | 0.277 | -0.003 0.001 |
| Decile 7 # Age | -0.003 | 0.000 | -0.005 -0.001 |
| Decile 8 # Age | -0.005 | 0.000 | -0.007 -0.004 |
| Decile 9 # Age | -0.010 | 0.000 | -0.012 -0.008 |
| Decile 10 # Age | -0.004 | 0.000 | -0.006 -0.003 |
| Intercept | 1.038 | 0.000 | 0.925 1.151 |
| RE parameters  site ID: identity  var(_cons)  var(residual) | 0.037(0.028, 0.050)  0.647( 0.641, 0.653) |  |  |
|  |  |  |  |
|  |  |  |  |
|  |  |  |  |

**Appendix II**

**Tertiary ICUs**


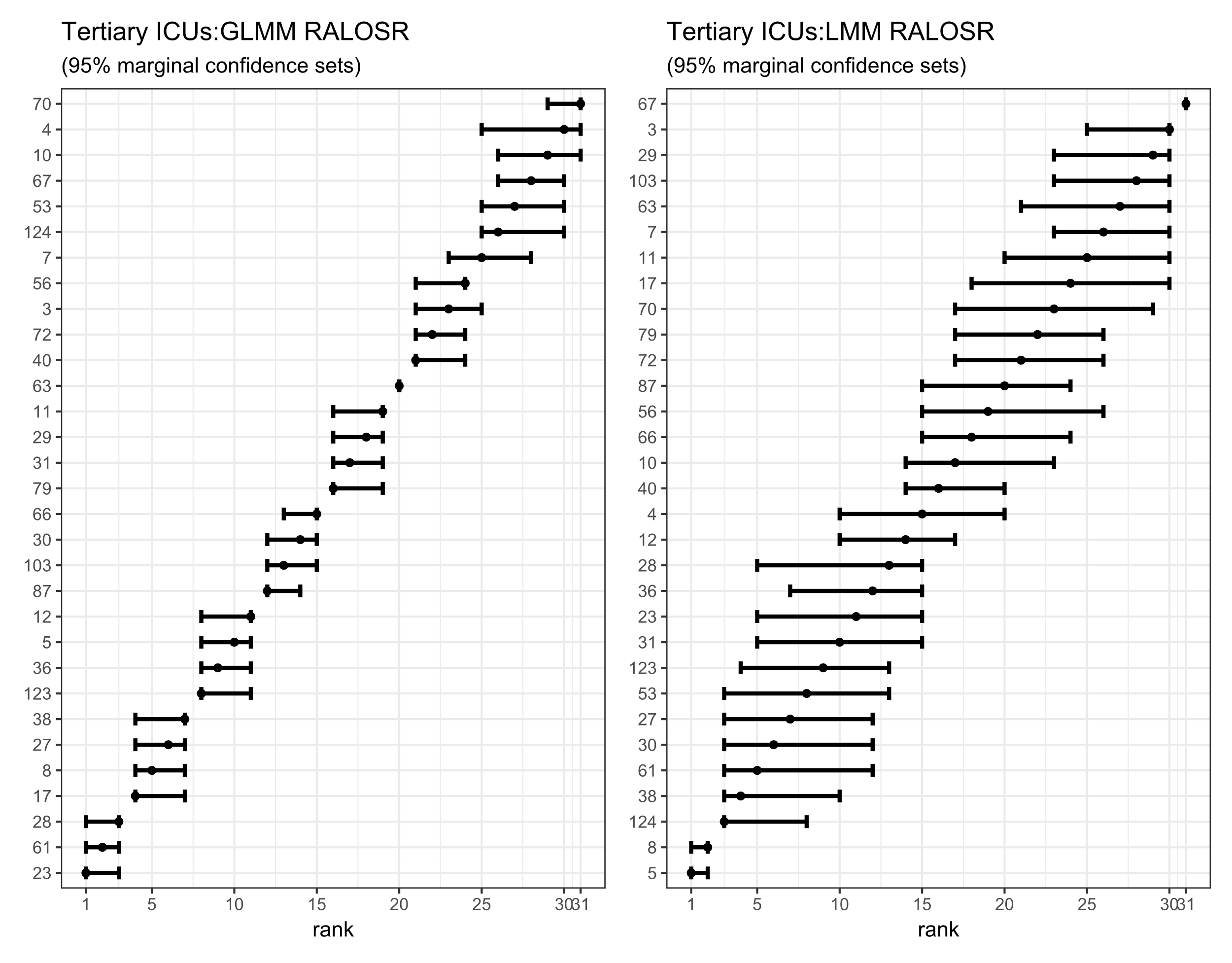


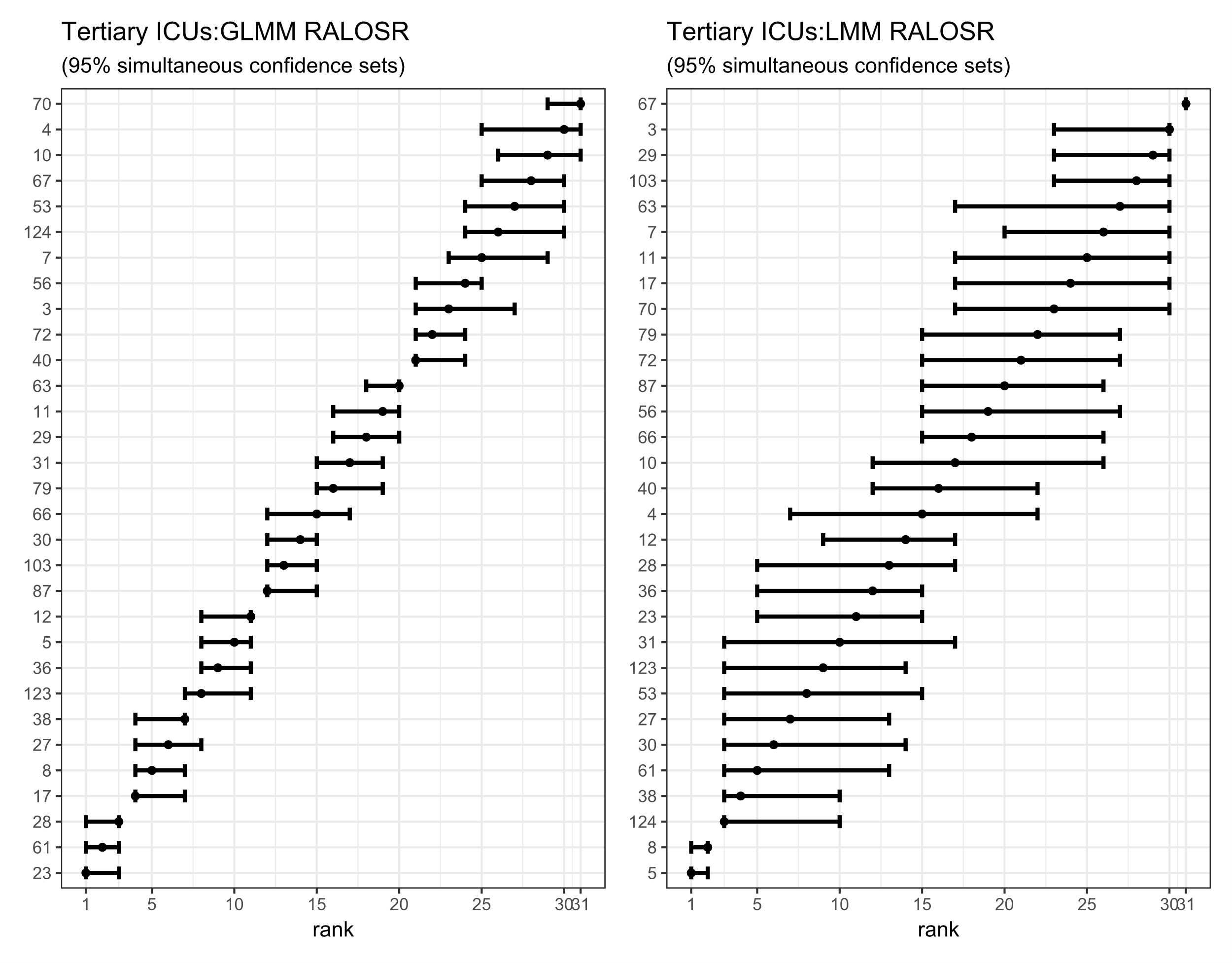


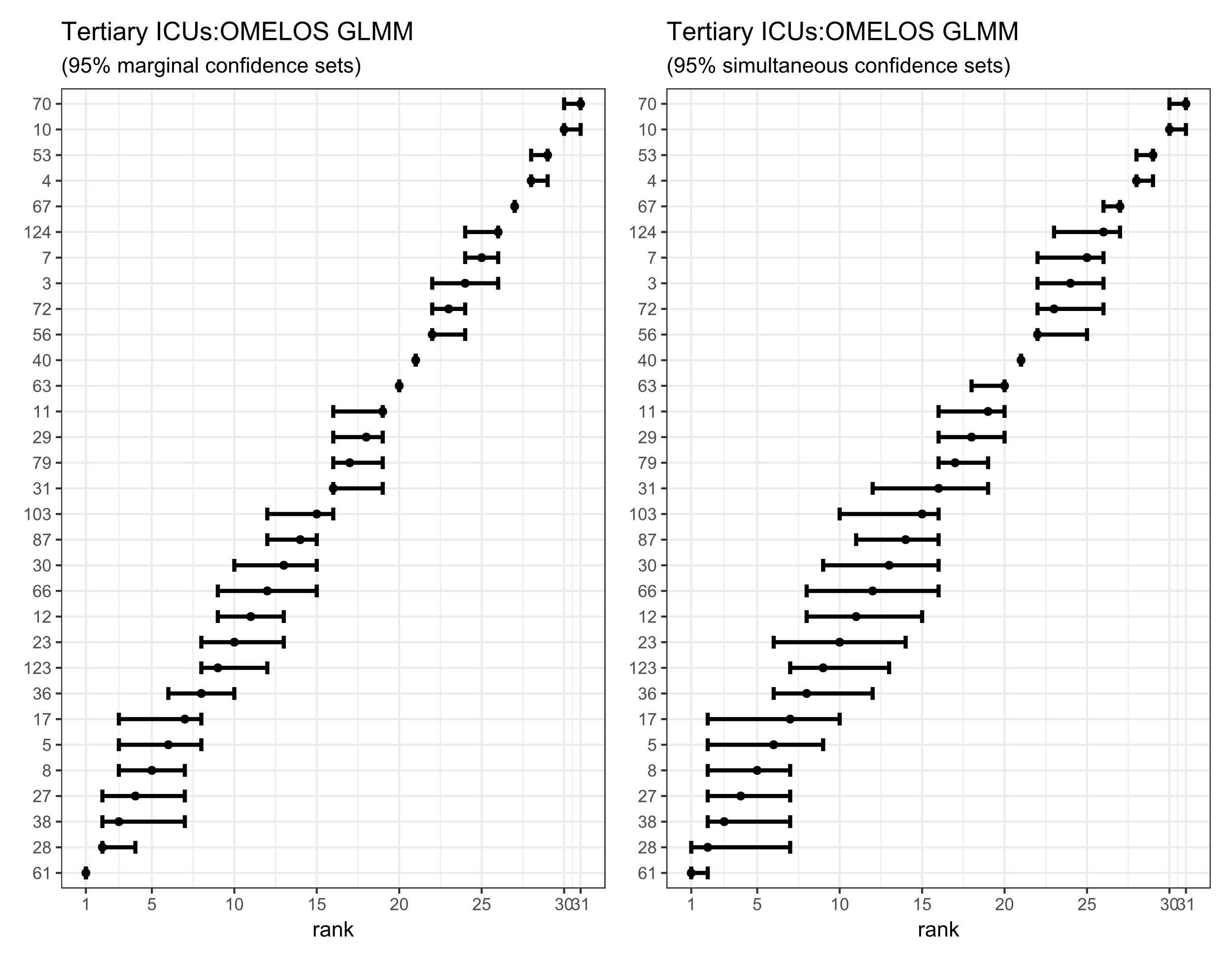


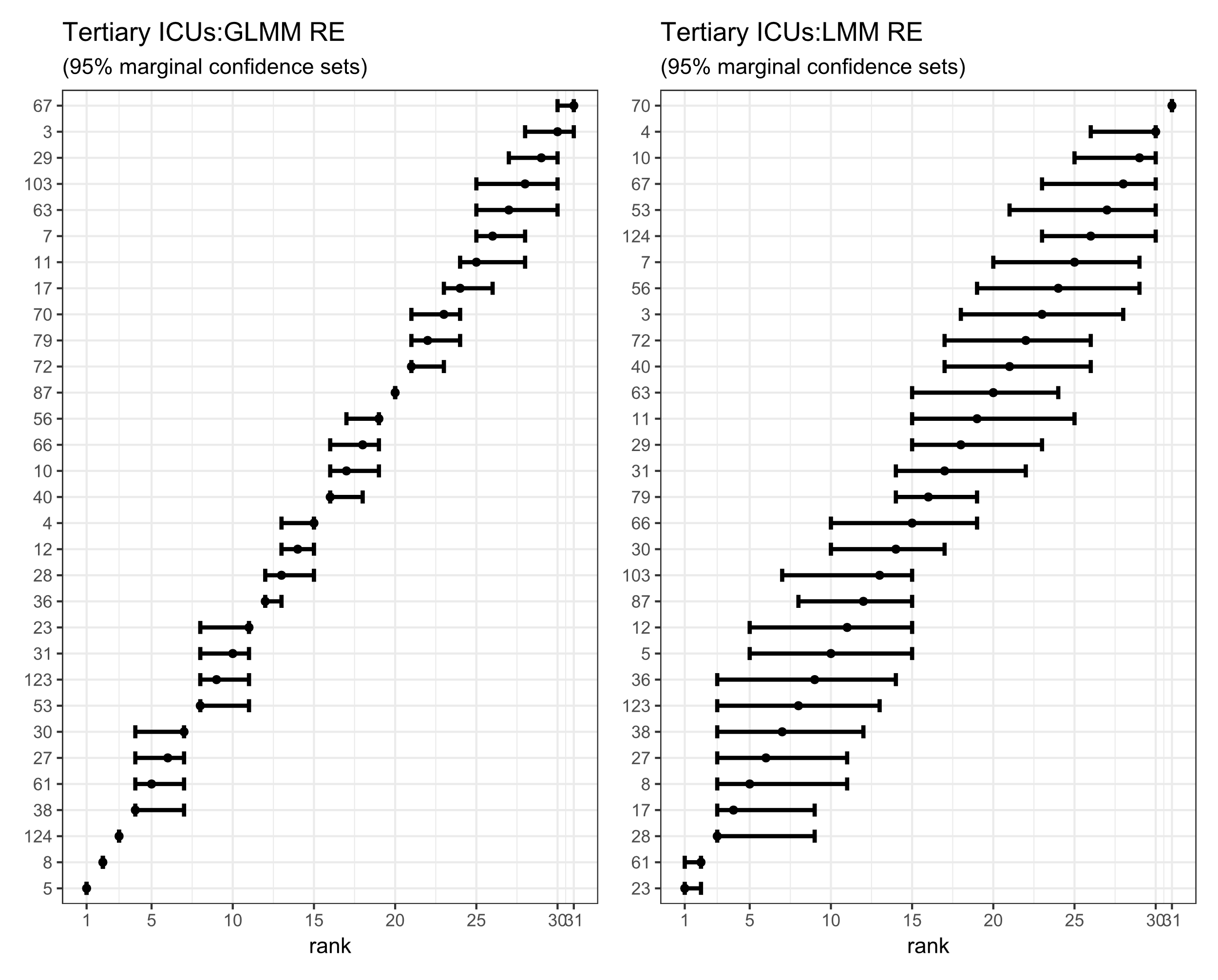


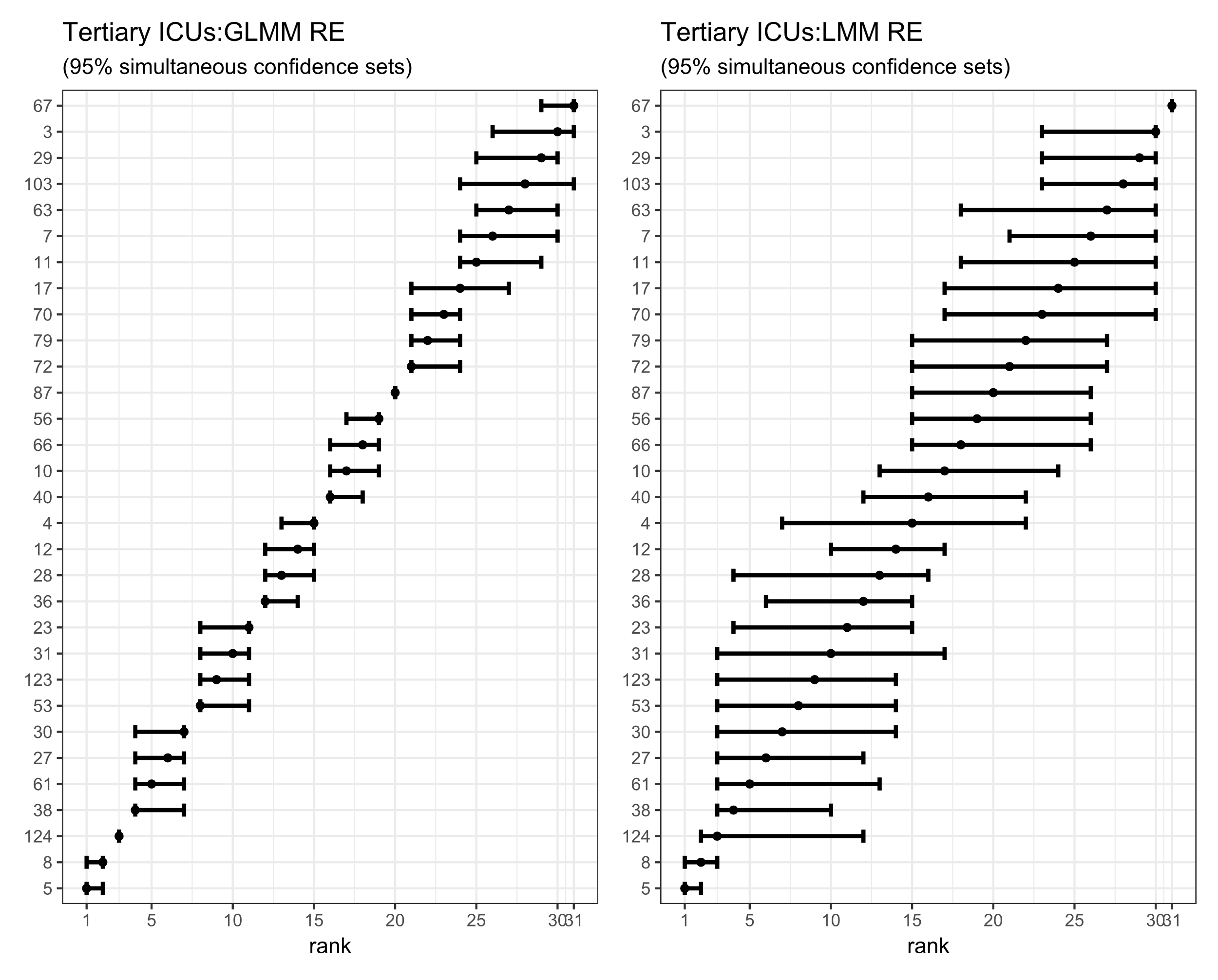


**Metropolitan ICUs**


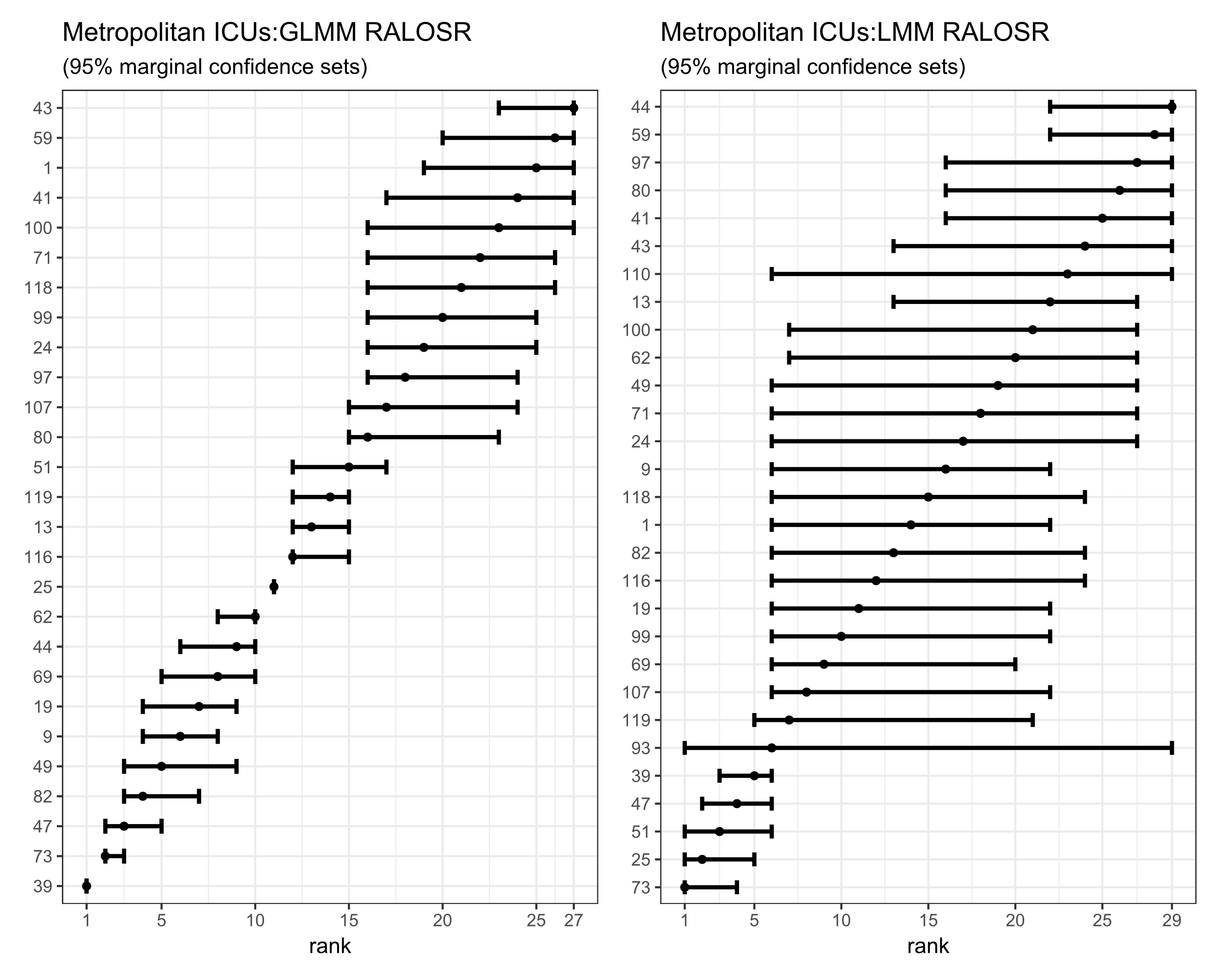


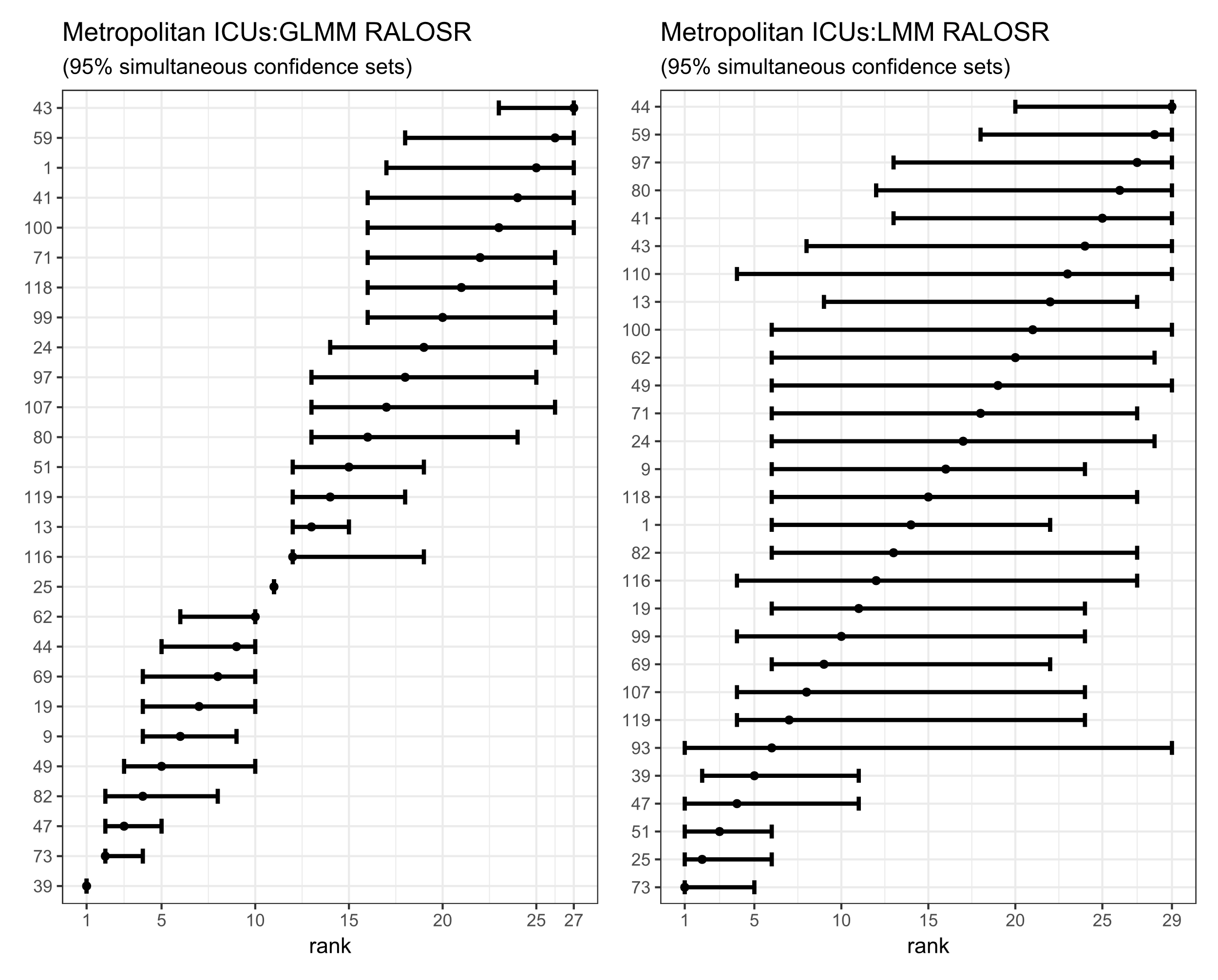


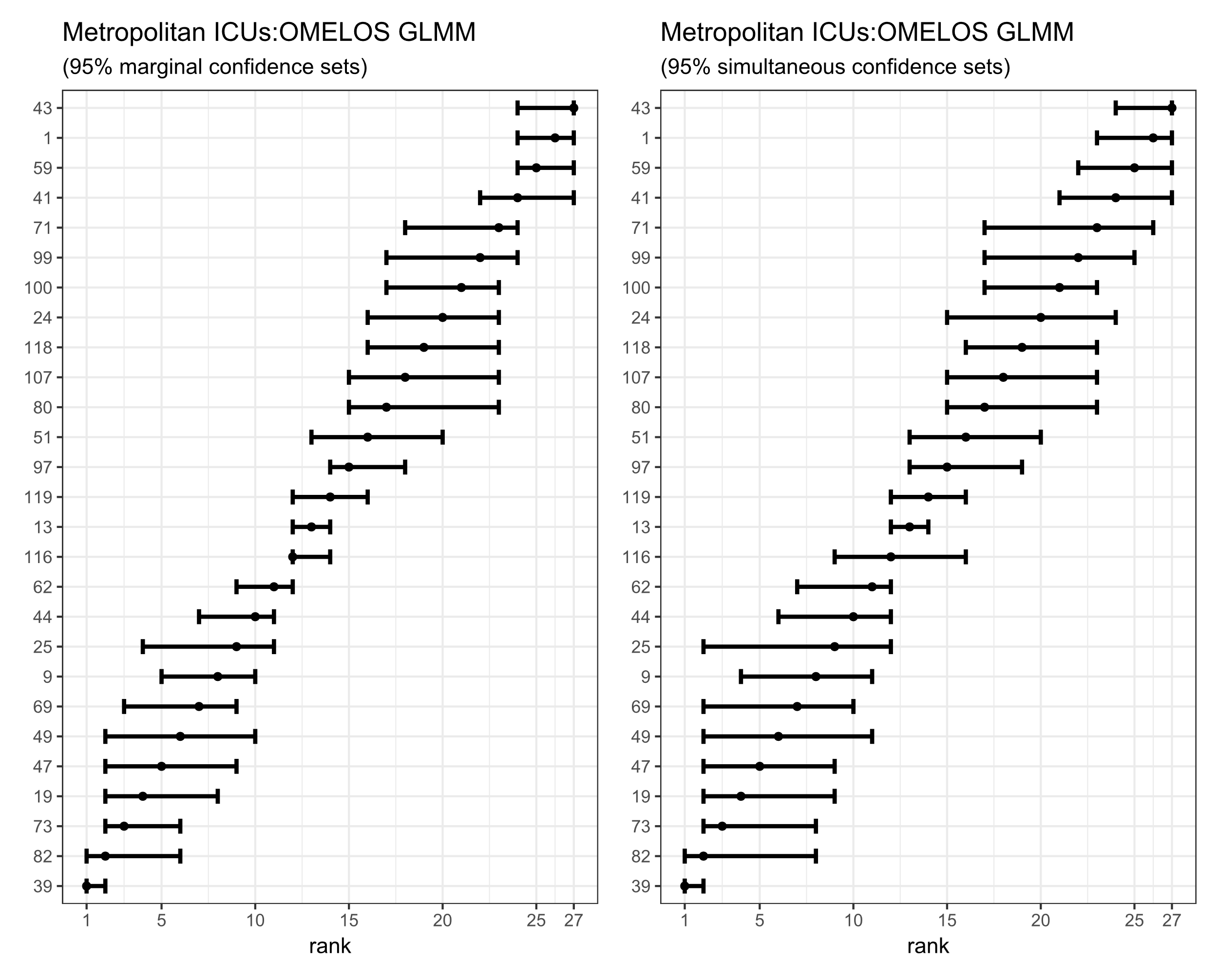


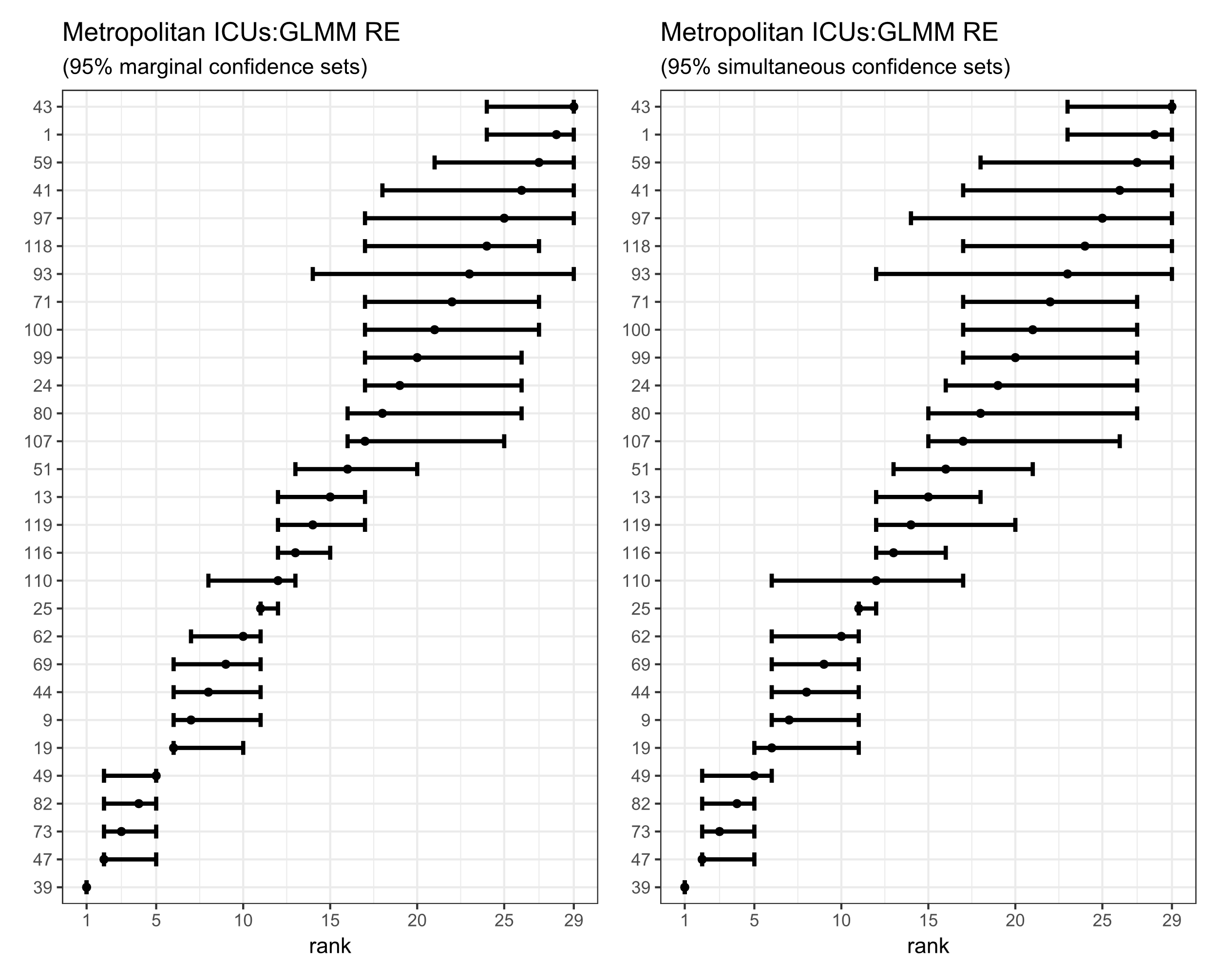


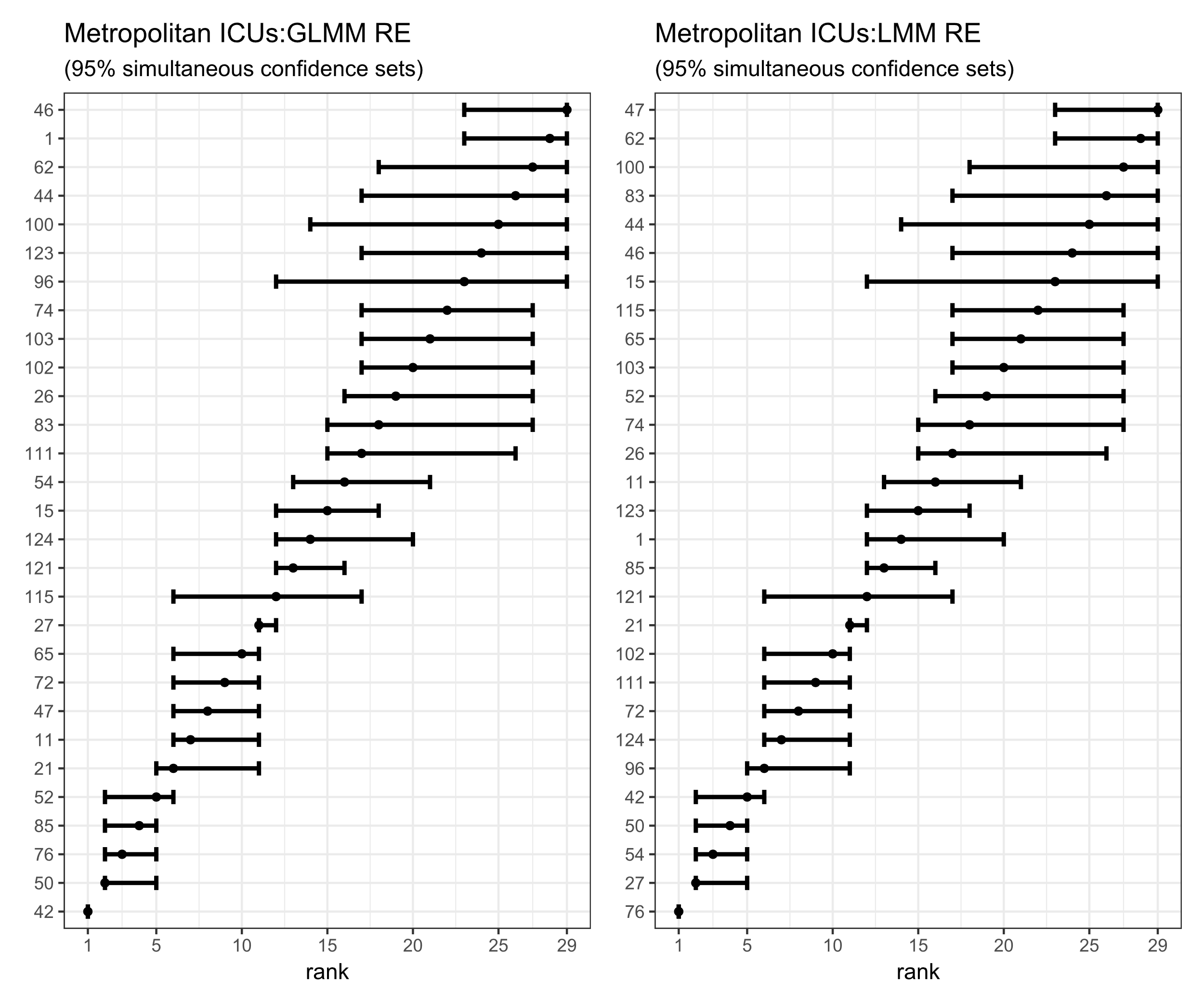


**Rural / Regional ICUs**


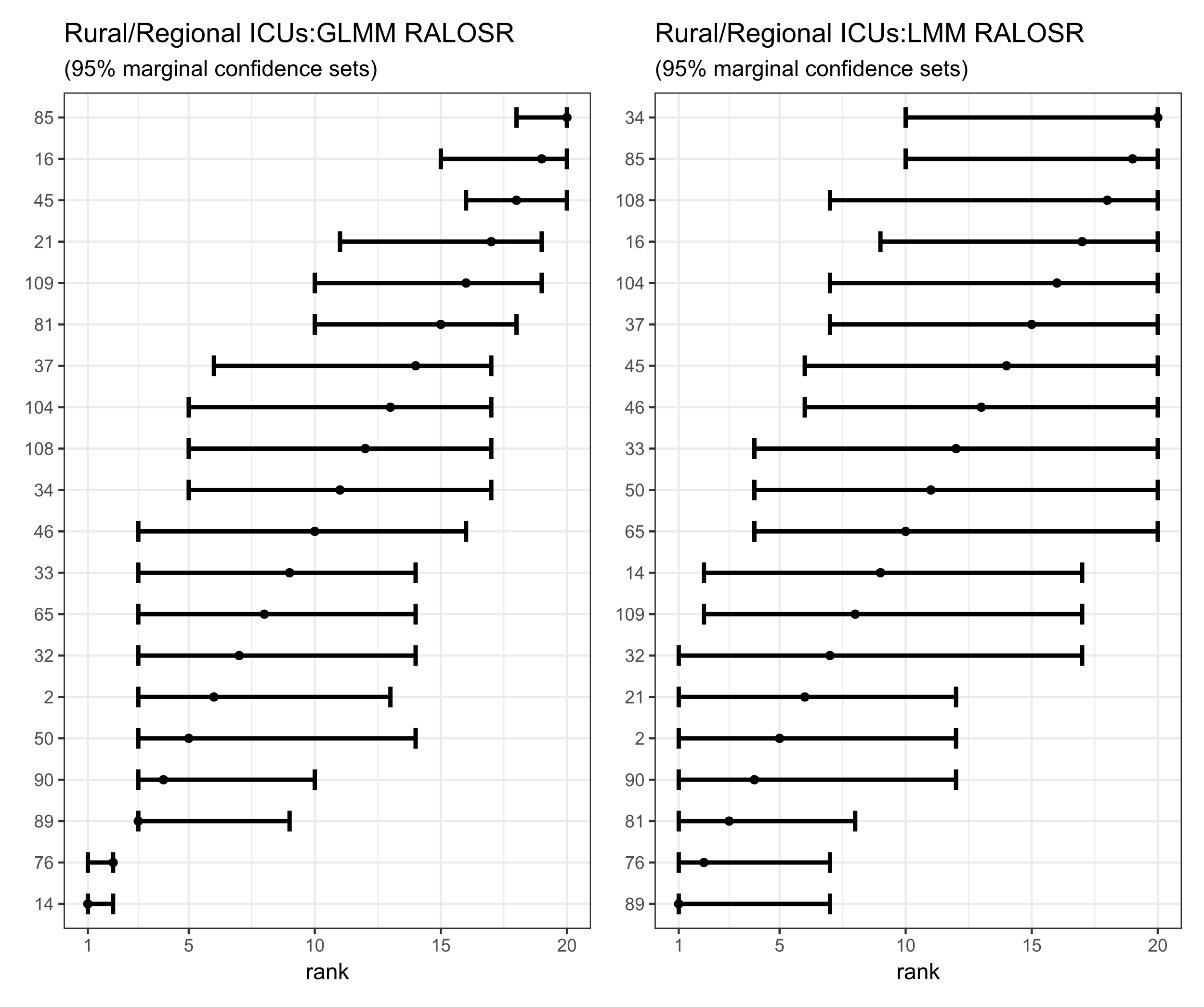


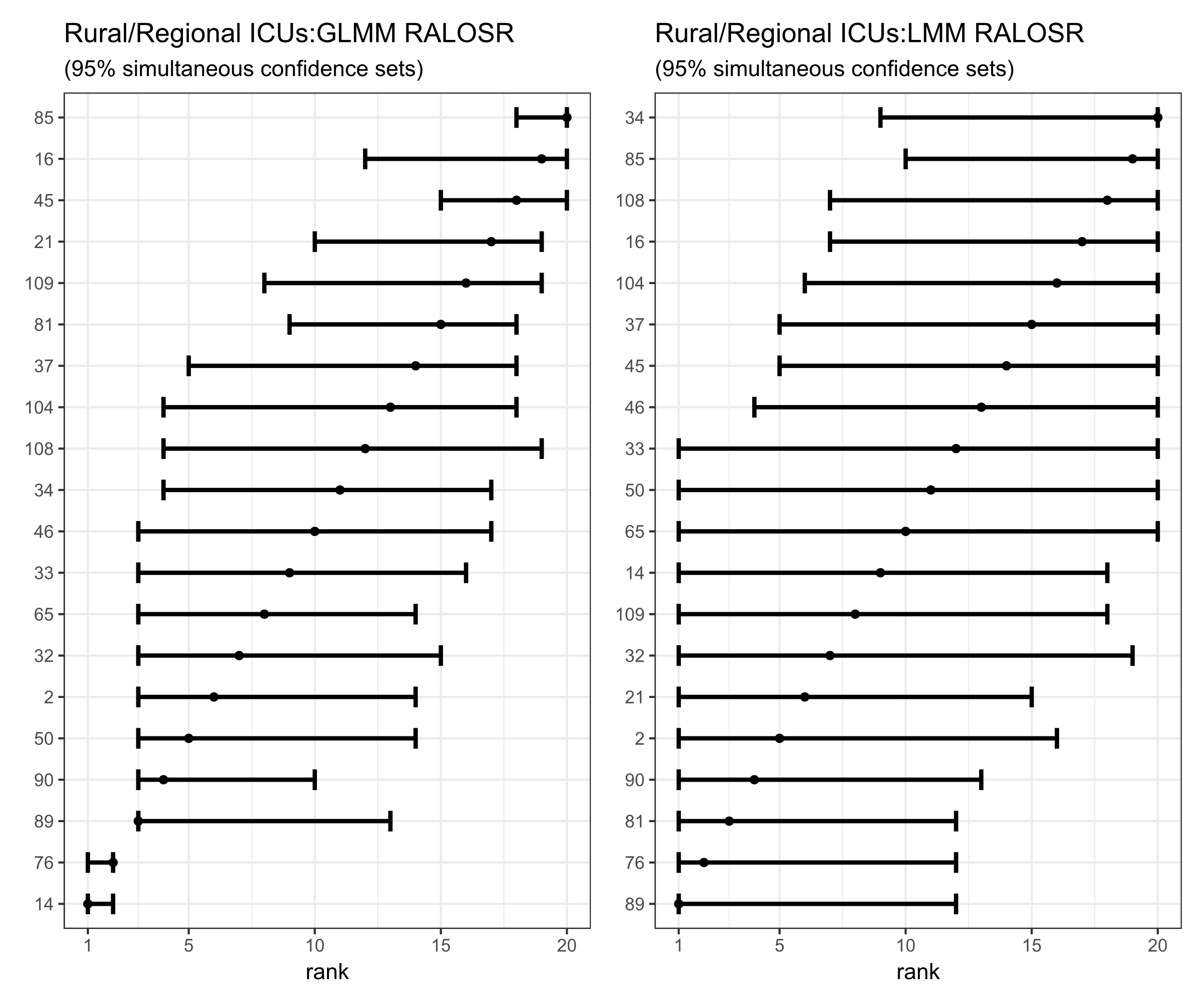


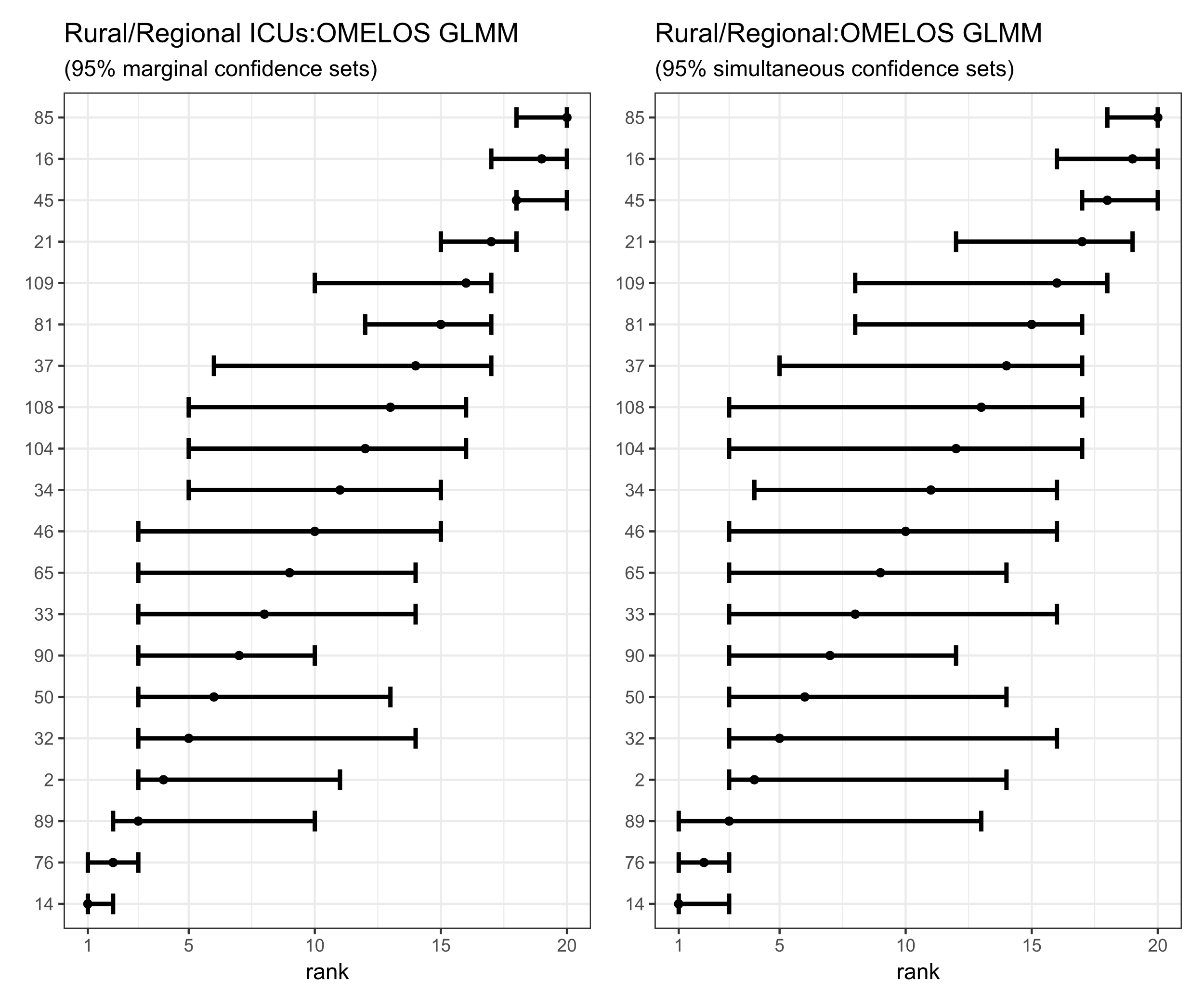


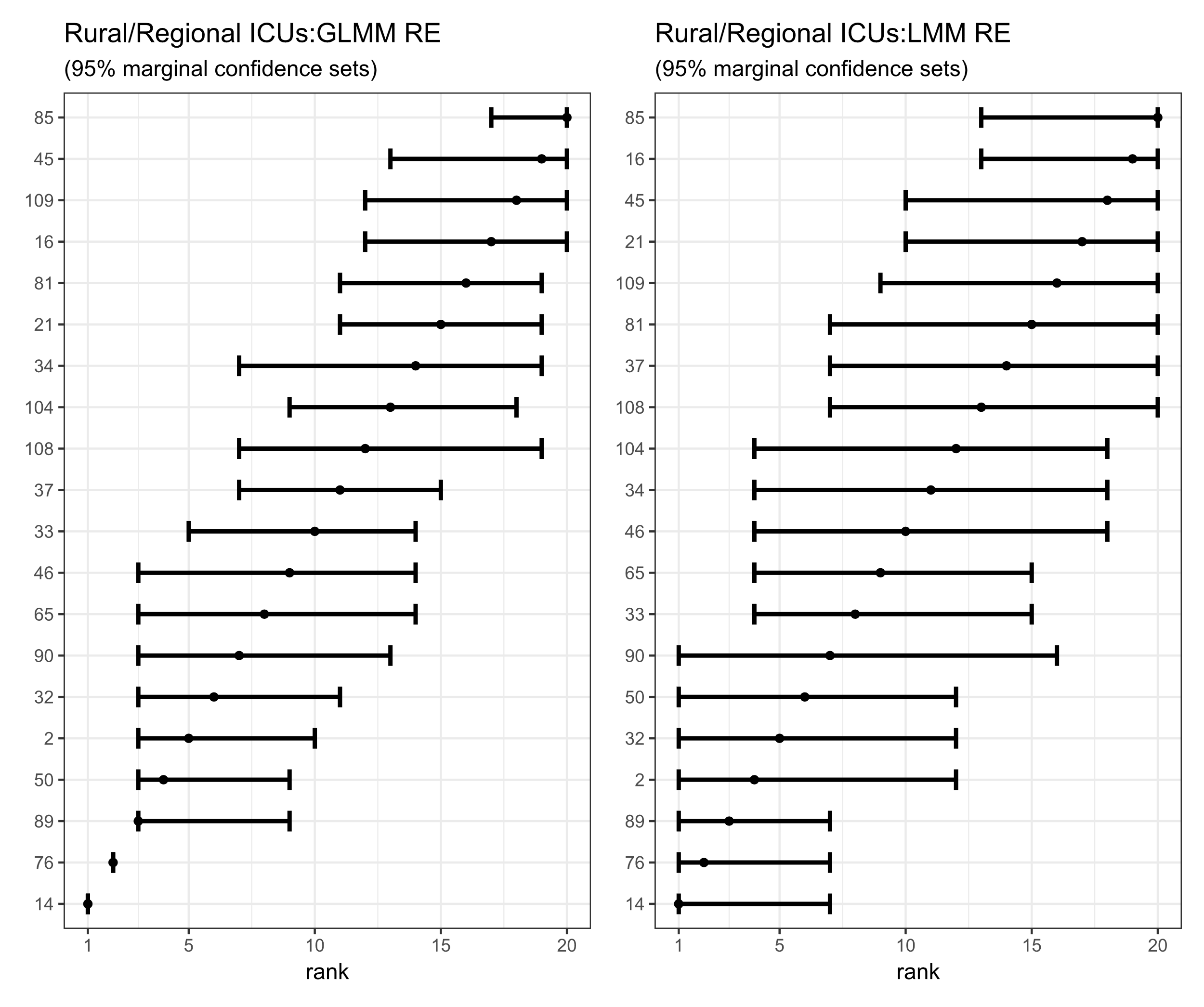


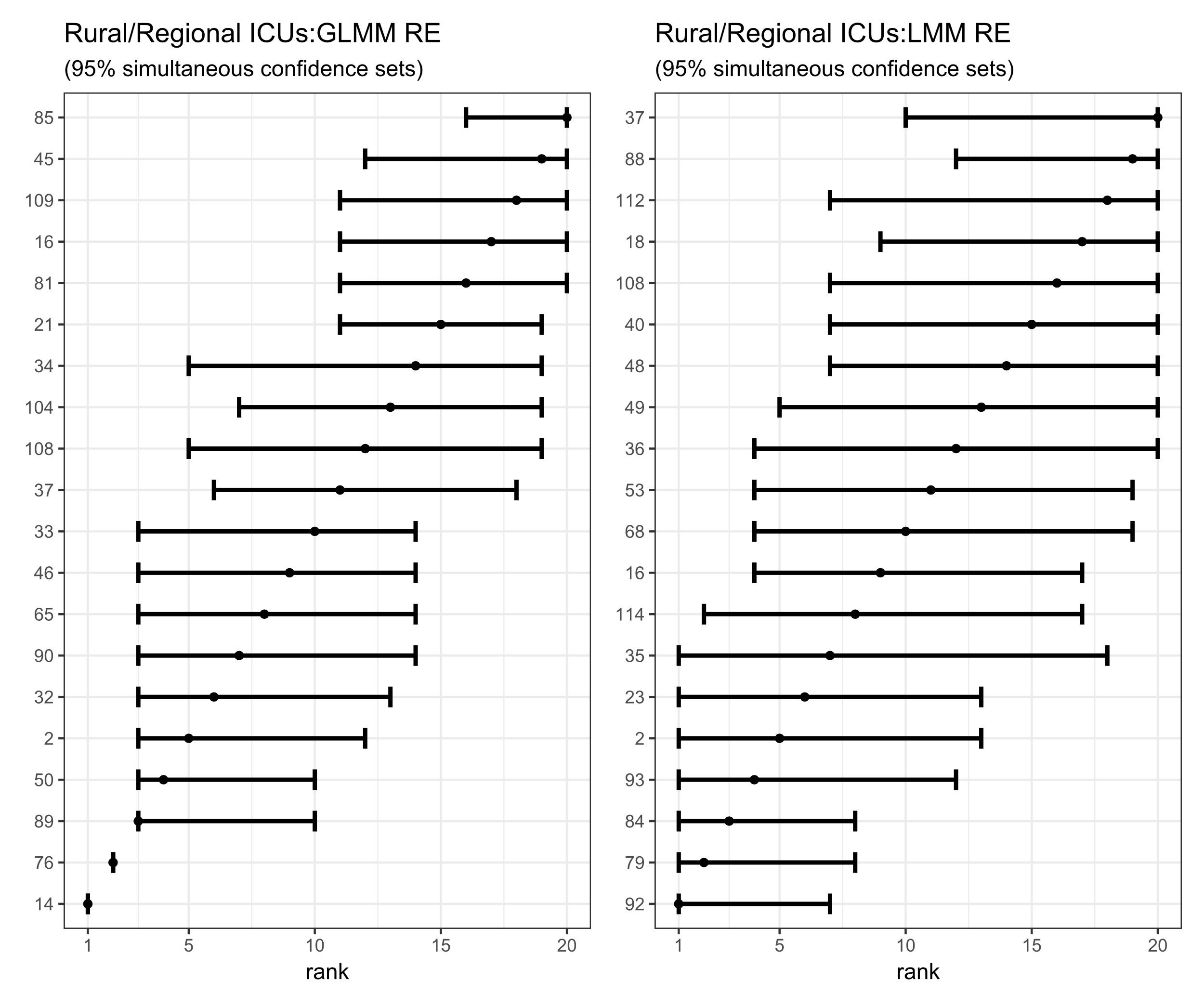


**Private ICUs**


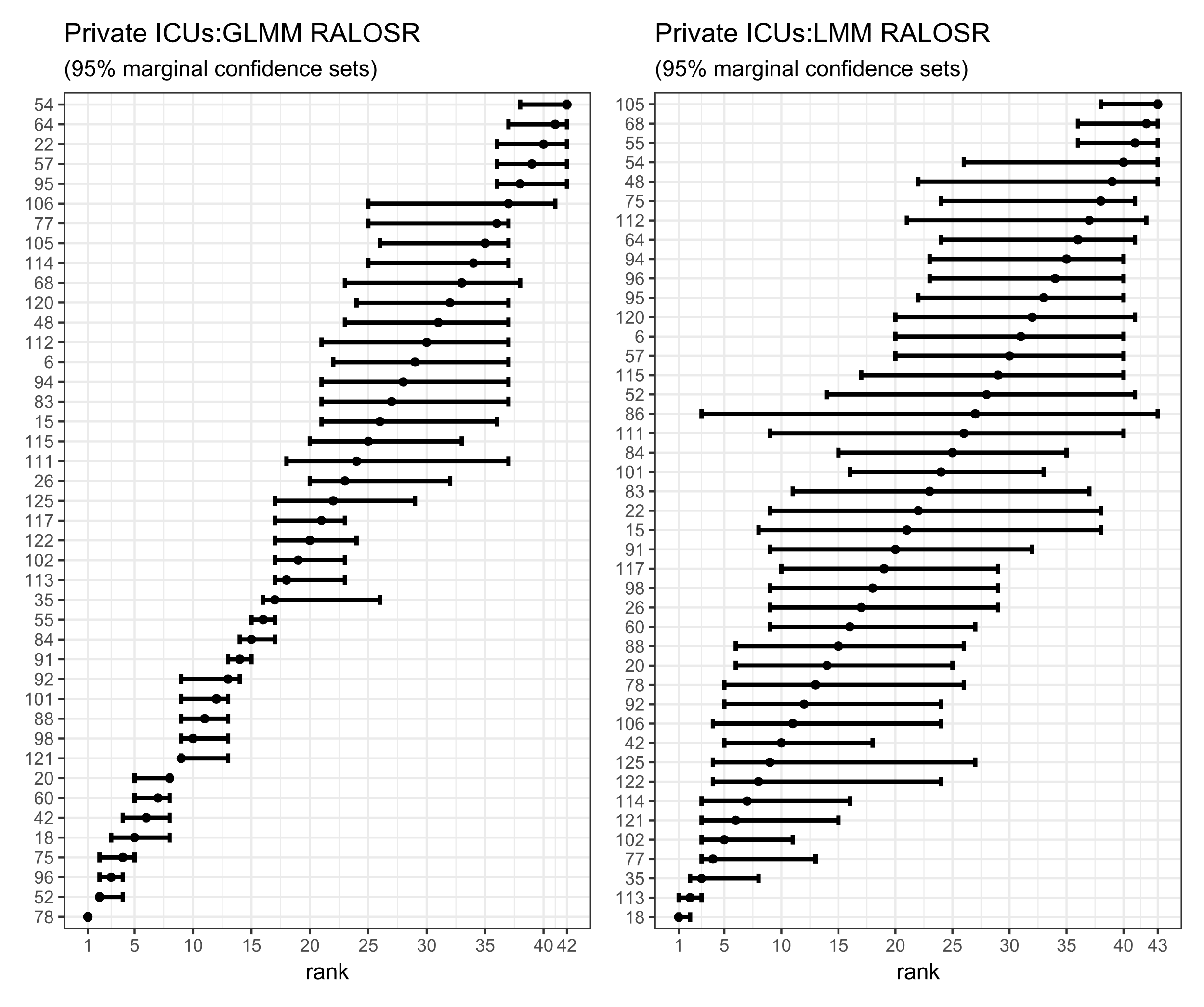


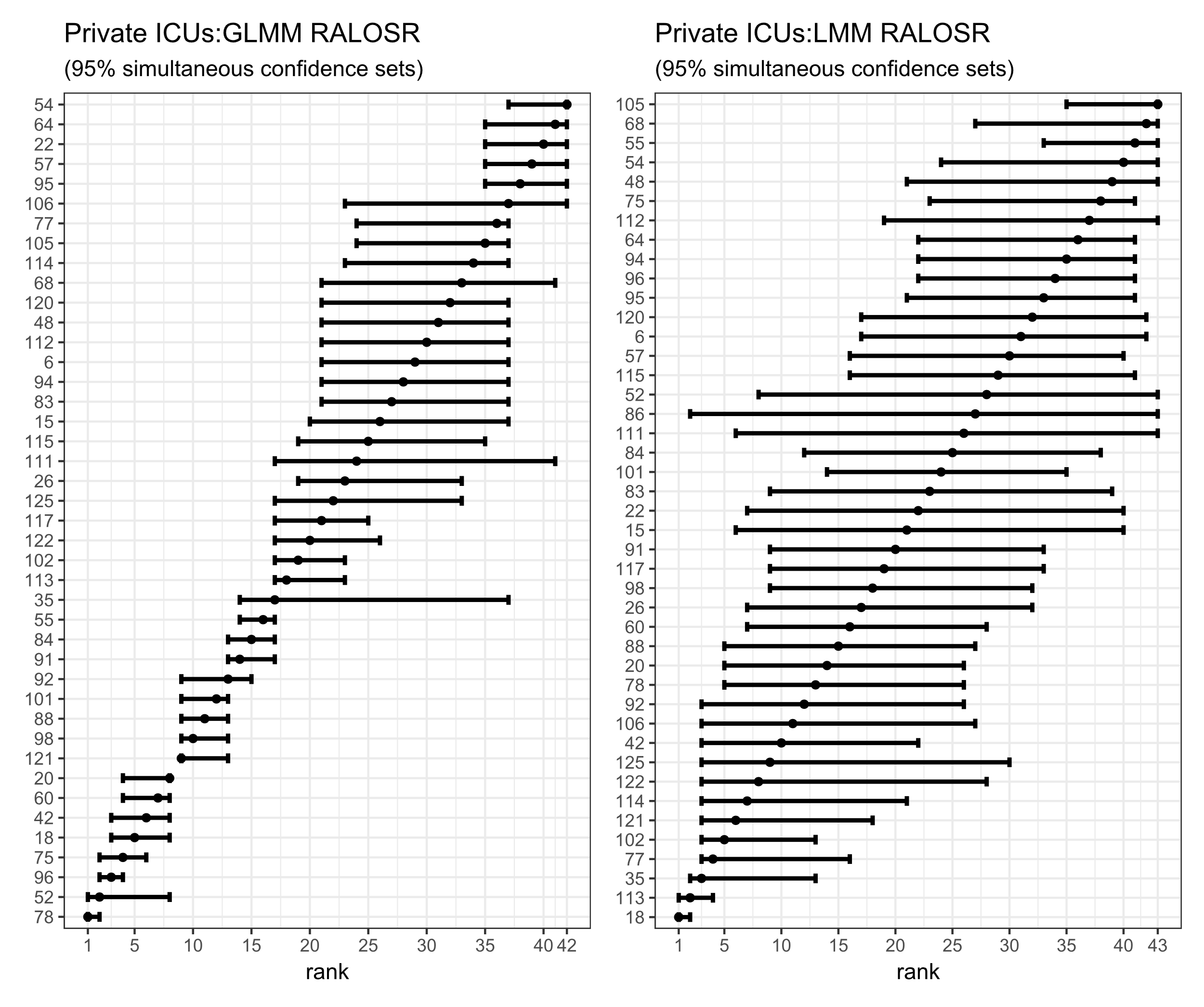


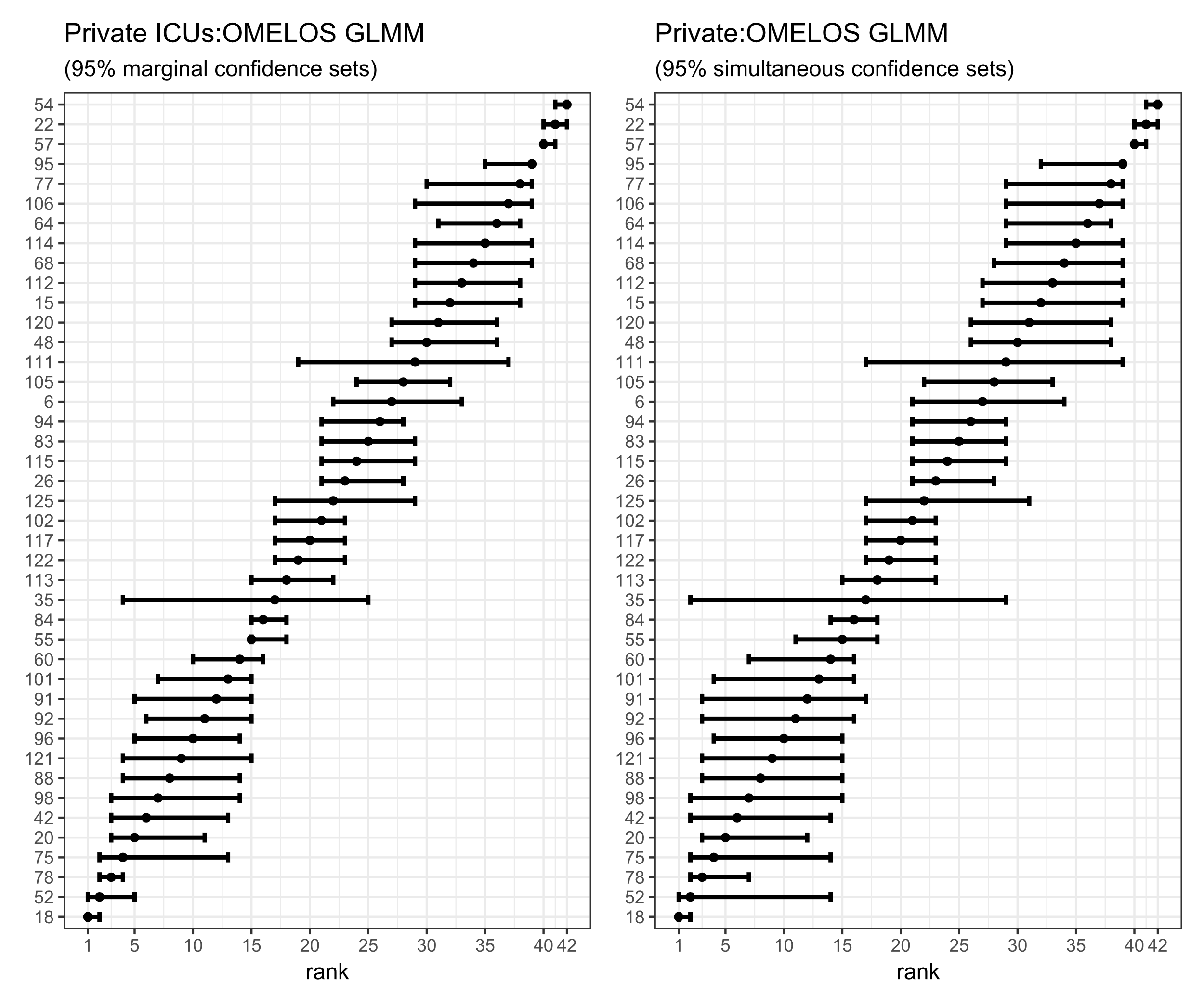


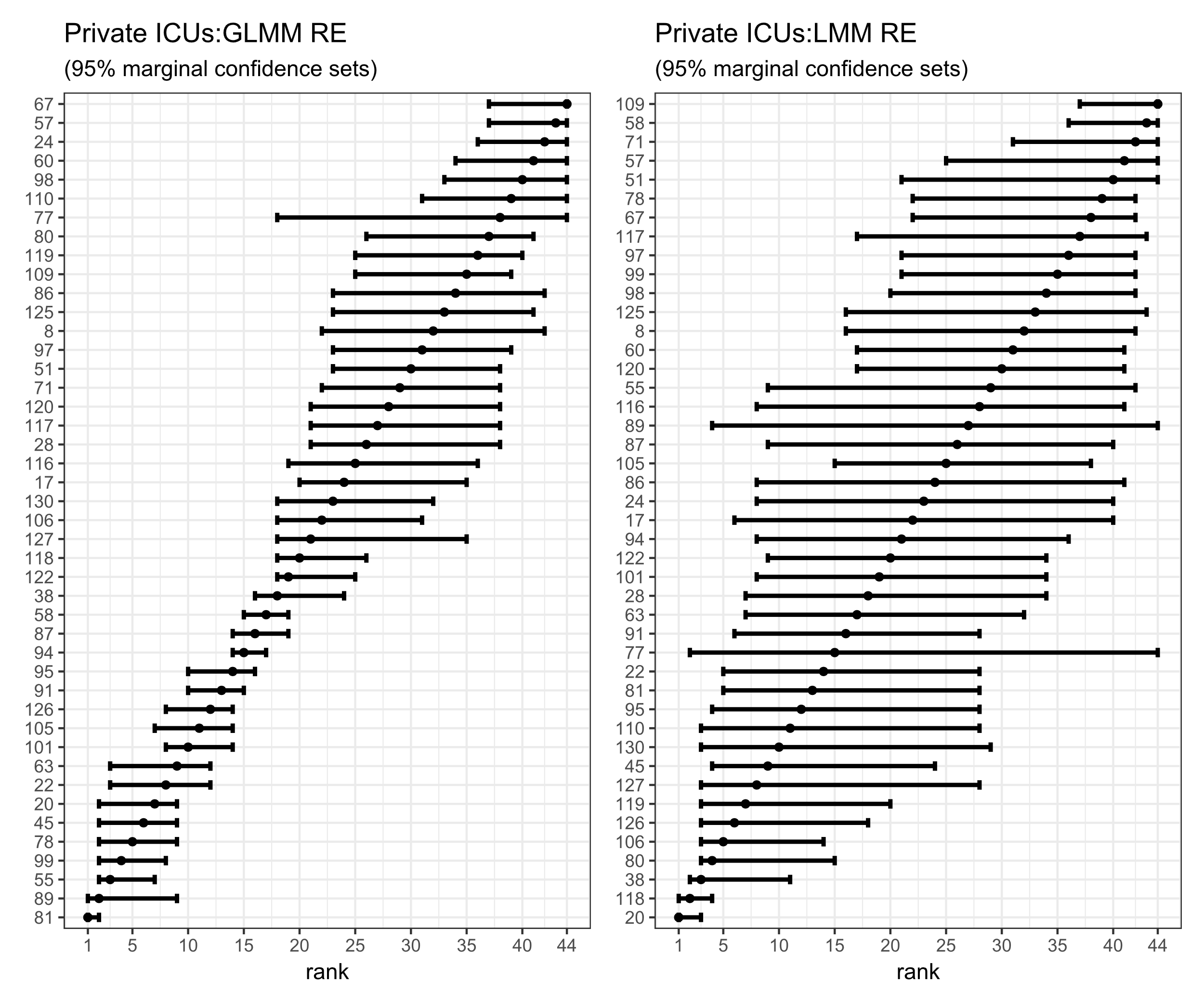


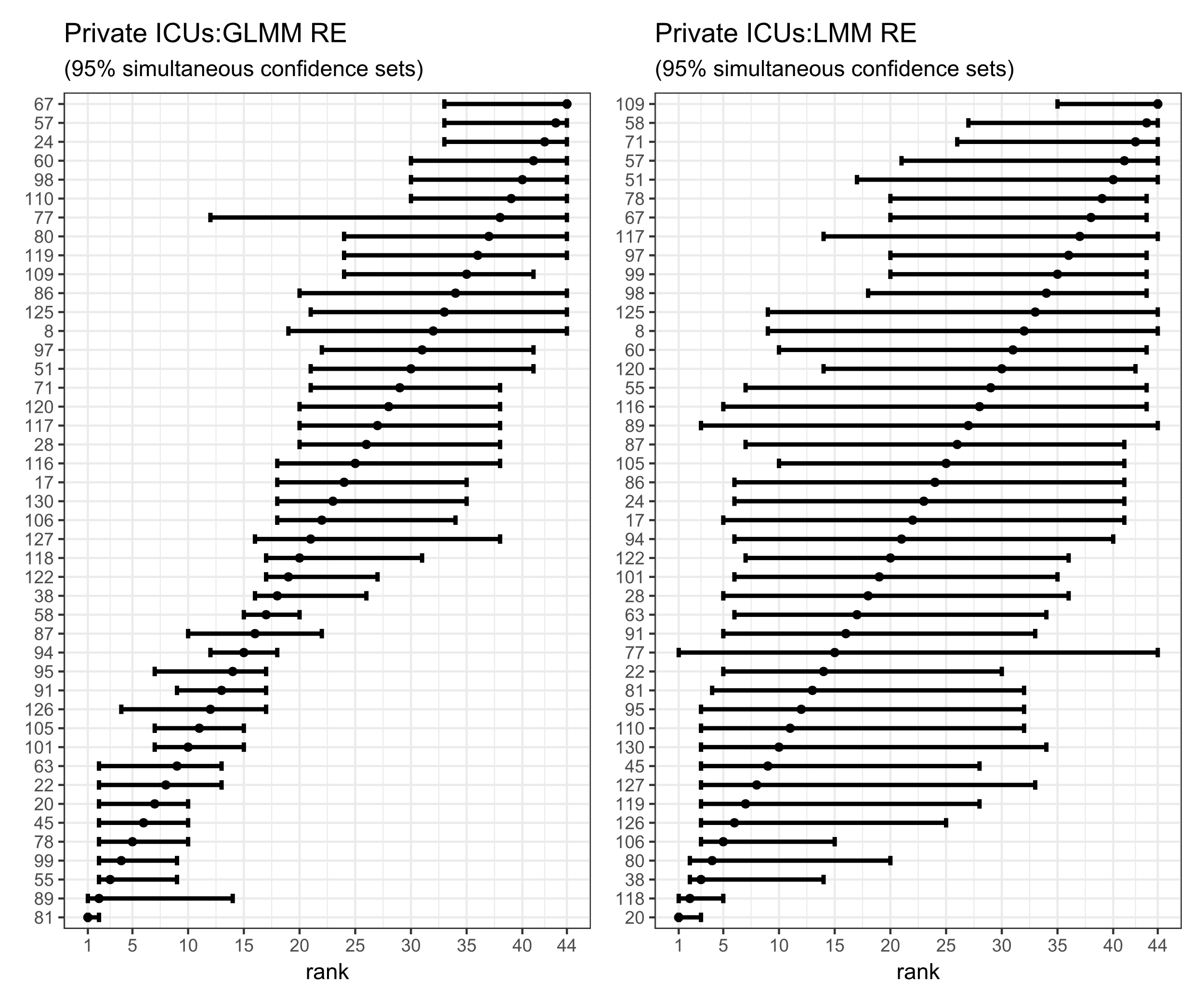


References

1. Kuha J: **AIC and BIC: Comparisons of Assumptions and Performance**. *Sociological Methods Research* 2004, **33**(2):188-229.

2. Knaus WA, Wagner DP, Draper EA, Zimmerman JE, Bergner M, Bastos PG, Sirio CA, Murphy DJ, Lotring T, Damiano A: **The APACHE III prognostic system. Risk prediction of hospital mortality for critically ill hospitalized adults**. *Chest* 1991, **100**(6):1619-1636.

3. Angus DC, Linde-Zwirble WT, Sirio CA, Rotondi AJ, Chelluri L, Newbold RC, III, Lave JR, Pinsky MR: **The effect of managed care on ICU length of stay: implications for medicare**. *JAMA* 1996, **276**(13):1075-1082.

4. Straney LD, Udy AA, Burrell A, Bergmeir C, Huckson S, Cooper DJ, Pilcher DV: **Modelling risk-adjusted variation in length of stay among Australian and New Zealand ICUs**. *Plos One* 2017, **12**(5).

5. StataCorp: **estatic—Display information criteria (Version 18)**. *Available @* [*https://wwwstatacom/manuals/restaticpdf*](https://wwwstatacom/manuals/restaticpdf) 2023.
